# Cancer immune therapy using engineered ‛tail-flipping’ nanoliposomes targeting alternatively activated macrophages

**DOI:** 10.1038/s41467-022-32091-9

**Published:** 2022-08-04

**Authors:** Praneeth R. Kuninty, Karin Binnemars-Postma, Ahmed Jarray, Kunal P. Pednekar, Marcel A. Heinrich, Helen J. Pijffers, Hetty ten Hoopen, Gert Storm, Peter van Hoogevest, Wouter K. den Otter, Jai Prakash

**Affiliations:** 1 Engineered Therapeutics, Department of Advanced Organ bioengineering and Therapeutics, TechMed Centre, University of Twente, Drienerlolaan 5, 7500AE Enschede, The Netherlands; 2 Multi-scale Mechanics, Thermal and Fluid Engineering, Faculty of Engineering Technology, University of Twente, Drienerlolaan 5, 7500AE Enschede, The Netherlands; 3 Physics and Physical Chemistry of Foods, Wageningen University, PO Box 17, 6700 AA Wageningen, The Netherlands; 4 Department of Pharmaceutics, Utrecht University, Universiteitsweg 99, 3584CG Utrecht, The Netherlands; 5 Department of Surgery, Yong Loo Lin School of Medicine, National University of Singapore, C/O NUHS Tower Block, Level 8. IE Kent Ridge Road, Singapore, Singapore; 6 Phospholipid Research Centre, Im Neuenheimer Feld 515, 69120 Heidelberg, Germany

**Keywords:** Nanotechnology in cancer, Cancer microenvironment, Monocytes and macrophages, Immunotherapy

## Abstract

Alternatively-activated, M2-like tumor-associated macrophages (TAM) strongly contribute to tumor growth, invasiveness and metastasis. Technologies to disable the pro-tumorigenic function of these TAMs are of high interest to immunotherapy research. Here we show that by designing engineered nanoliposomes bio-mimicking peroxidated phospholipids that are recognised and internalised by scavenger receptors, TAMs can be targeted. Incorporation of phospholipids possessing a terminal carboxylate group at the *sn-2* position into nanoliposome bilayers drives their uptake by M2 macrophages with high specificity. Molecular dynamics simulation of the lipid bilayer predicts flipping of the *sn-2* tail towards the aqueous phase, while molecular docking data indicates interaction of the tail with Scavenger Receptor Class B type 1 (SR-B1). In vivo, the engineered nanoliposomes are distributed specifically to M2-like macrophages and, upon delivery of the STAT6 inhibitor (AS1517499), zoledronic acid or muramyl tripeptide, these cells promote reduction of the premetastatic niche and/or tumor growth. Altogether, we demonstrate the efficiency and versatility of our engineered “tail-flipping” nanoliposomes in a pre-clinical model, which paves the way to their development as cancer immunotherapeutics in humans.

## Abstract (web summary)

Tumor-associated macrophages are mostly pro-tumorigenic, due to their re-programming by the tumor microenvironment. Here authors show that nanoliposomes, incorporating phospholipids with a flipping-tail chain, are engulfed specifically by intratumoral, alternatively activated macrophages, while delivering a cargo that converts these cells into anti-tumor macrophages.

## Introduction

Advances in the field of immuno-oncology have provided new opportunities for the treatment of cancer^1–3^. Harnessing tumor immune cells to attack cancer cells by modulating them within the tumor microenvironment (TME) is an attractive new option to combat cancer^4–6^. The TME is enriched with a variety of immune cells such as tumor-associated macrophages (TAM), T-lymphocytes, natural killer cells as well as other cells including cancer-associated fibroblasts, pericytes, and tumor vasculature cells^1,7^. TAMs are one of the most crucial immune cells which can account for up to 50% of solid tumors. TAMs play a pivotal role in inducing tumor progression, metastasis and resistance to therapy^8–10^. In general, macrophages are broadly classified into classically-activated M1-like macrophages (pro-inflammatory and anti-tumoral) and alternatively-activated M2-like macrophages (anti-inflammatory and pro-tumoral) phenotypes^8,11,12^. However, some studies showed that TAM subsets exist beyond M1 and M2 phenotypes^13,14^. Macrophages polarized with Th1 cytokines such as interferon-gamma (IFNγ), interleukin-12 (IL-12) or bacterial lipopolysaccharide (LPS), or Toll-like agonists acquire M1-like state and secrete pro-inflammatory cytokines (IL-1β, IL-6, IL-12, tumor necrosis factor-alpha (TNF-α)). In contrast, with the exposure to Th2 cytokines such as IL-4, IL-13, colony stimulating factor-1 (CSF-1) leads to the M2-like state, which leads to secretion of anti-inflammatory cytokines (IL-10, transforming growth factor beta (TGF-β)). In tumors, TAMs are either recruited from the bone marrow or originating from the macrophages already residing in the tumor^8,11,15^. During tumorigenesis, TAMs acquire proliferative capacity after differentiation via cytokines and growth factors secreted by cancer cells and other cells in the TME. TAMs are highly plastic and can rapidly adapt to the perturbations arising in the microenvironment and can express both M1 and M2 markers. M2-like TAMs represent the pro-tumorigenic phenotype which are able to stimulate the tumor cell growth via their large secretome of cytokines and support angiogenesis, immunosuppression, invasion, and metastasis^15^. Therefore, specific deactivation, elimination of M2-TAMs and their re-programming into anti-tumoral M1 type are attractive strategies to hamper TAM-driven pro-tumorigenic processes.

In the past years, there has been a strong focus on the modulation of TAMs to inhibit their pro-tumorigenic functions^16–19^. Depleting the macrophages or blocking the recruitment of monocytes using bisphosphonates or colony stimulating factor receptor (CSF-1) inhibitors, respectively led to inhibition of tumor growth and metastasis^20,21^. Recently, we showed that targeting the STAT6 pathway with a small molecule, AS1517499, hampered M2 differentiation of TAMs and reduced M2 markers, and tumor migration. Interestingly, treatment with high dose of AS1517499 attenuated the early metastasis formation in a 4T1 orthotopic tumor model^22^.

In literature, several strategies have been proposed to target M2 macrophages mainly via the mannose receptor (CD206, MRC-1) using either mannose ligand or using an antibody or peptide against the receptor^23–26^. Macrophages have been shown to express several phagocytic receptors including scavenger receptors to sense and recognize pathogen-associated molecular patterns, oxidized phospholipids and dead cells^27^. Previously, we have studied the phagocytic behavior of M1 and M2 macrophages in engulfing silica nanoparticles and identified several phagocytic receptors overexpressed specifically by M2 macrophages using phagocytosis-related gene array^28^. The M2-upregulated phagocytic receptors included a family of scavenger receptors i.e., class A (COLEC12) and class B (CD36, Scavenger Receptor Class B type 1 (SR-B1))^29,30^. COLEC12 displays several functions associated with host defense by promoting binding and phagocytosis of bacteria and yeast. Both COLEC12 and CD36 are involved in binding to oxidized low-density lipoprotein (oxLDL) containing fatty acid products with terminal carboxylate groups^30–32^. SR-B1 is involved in the engulfment of apoptotic cells by recognizing the oxidized phospholipids which are displayed on cell membranes upon apoptosis^32^. The carboxylated phospholipids are a type of oxidized phospholipids which are generated from peroxides derived from (partially) oxidized (poly)unsaturated diacyl- and alk(en)ylacyl glycerophospholipids.

In this study, we harness this biomimicry process of recognition of carboxylated phospholipids by these specific scavenger receptors to develop a novel strategy to target M2-TAMs. We prove that these carboxylated phospholipids can flip their *sn-2* tail and specifically engages with scavenger receptor SR-B1. Then, we confirm that nanoliposomes distribute to M2-TAMs in vivo and delivery of different compounds (STAT6 inhibitor (AS1517499), zoledronic acid or muramyl tripeptide) reduces the premetastatic niche and/or tumor growth in vivo. Altogether, our engineered “tail-flipping” nanoliposomes are in potential to be developed as cancer immunotherapeutics as well as may be used for other diseases with M2 polarization including asthma and fibrosis^33^.

## Results

### Nanoliposomes targeting M2 type macrophages in vitro

**Fig. 1:**
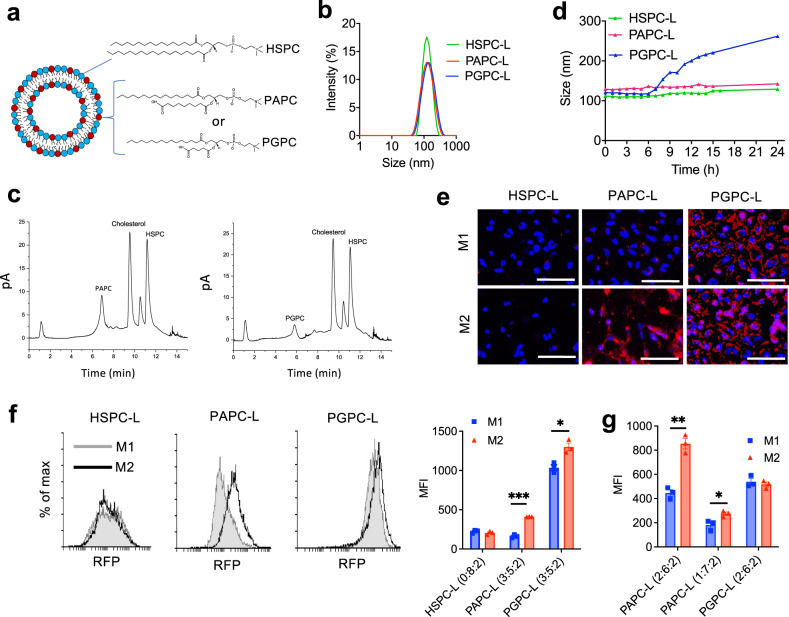
Characterization and uptake of M2-targeted nanoliposomes. **a** Representative illustration of nanoliposomes showing incorporation of HSPC, PAPC and PGPC phospholipid. **b** Typical histogram showing the size distribution of HSPC nanoliposomes (HSPC-L, HSPC:Cholesterol = 8:2), PAPC-L (PAPC:HSPC:Cholesterol = 3:5:2) and PGPC-L (PGPC:HSPC:Cholesterol = 3:5:2) obtained from dynamic light scattering method. **c** Typical chromatogram of lipid mixtures isolated from PAPC-L and PGPC-L, analyzed using ultra-high performance liquid chromatography (uHPLC) with corona charged aerosol detector (CAD). **d** Stability analysis of nanoliposomes using size measurement in culture media at 37 °C during 24 h. **e**–**g** Representative fluorescent images of cellular uptake of 1,1′-dioctadecyl-3,3,3′,3′-tetramethylindocarbocyanine (DiI)-containing HSPC-L, PAPC-L and PGPC-L by M1 and M2 differentiated macrophages from THP-1 monocytes at *t* = 2 h. Blue: DAPI, Red: nanoliposomes labeled with DiI, scale bar = 50 µm. **f** Representative flow cytometry histograms and liposomal uptake (mean fluorescent intensity (MFI)) of HSPC-L, PAPC-L (3:5:2) or PGPC-L (3:5:2) by M1 and M2 macrophages after incubation for 2 h (left to right: ***p = 0.000037, *p = 0.012). **g** Liposomal uptake (MFI) of PAPC-L (2:6:2, 1:7:2) or PGPC-L (2:6:2) by M1 and M2 macrophages after incubation for 2 h (left to right: **p = 0.0013, *p = 0.031). Data represent the mean + standard error of the mean (SEM) from three independent experiments. Statistical analysis was performed with Multiple unpaired *t*-tests with correction for multiple comparisons using the Holm–Sidak’s method. Source data are provided as a Source Data file.

We used stable and saturated phospholipids containing a *sn-2* fatty acid chain with a terminal carboxyl group, i.e., 1-palmitoyl-2-azelaoyl-sn-glycero-3-phosphocholine (PAPC) and 1-palmitoyl-2-glutaryl-sn-glycero-3-phosphocholine (PGPC), which are formed naturally from polyunsaturated fatty acids (Supplementary Fig. 1) . The details on the selection process of these lipids are explained in Supplementary Fig. 1. PAPC and PGPC phospholipids were incorporated into nanoliposomes at different molar ratios (2:6:2 or 3:5:2 mol: mol: mol; PAPC/PGPC:HSPC:Cholesterol) (Fig. 1a). The prepared nanoliposomes ranged from 80 to 116 nm (PDI ≤ 0.2) with the zeta potential ranging from −17 to −27.8 mV (Supplementary Table 1, Fig. 1b). The incorporation of different lipids was confirmed with ultra-high performance liquid chromatography (uHPLC) coupled to a corona charged aerosol detector (CAD) detector. The uHPLC chromatogram shows that HSPC-nanoLiposomes (HSPC-L) and PAPC-L maintained the initial lipid ratios, while PGPC-L had a lower incorporation (24% PGPC instead of 30%) (Fig. 1c, Supplementary Table 2). All formulations remained stable at 4 °C in PBS for 3 weeks with no significant changes (Supplementary Table 3). Furthermore, HSPC-L and PAPC-L showed high stability i.e., no change in size and no leakage of 1,1′-dioctadecyl-3,3,3′,3′-tetramethylindocarbocyanine (DiI) dye at 37 °C for up to 24 h in RPMI-1640 cell culture medium, while PGPC-L showed a gradual swelling in size within 24 h (Fig. 1d). This could be attributed to the weakly packed PGPC into the nanoliposomes.

To evaluate the uptake by different subsets of macrophages, we first differentiated human THP-1 monocytes into M1 and M2 macrophages using specific cytokines (M1: LPS + IFNγ; M2: IL4 + IL13). The successful differentiation was confirmed with the induced mRNA expression levels of specific markers (M1: TNF-α and IL-β; M2: DC-SIGN and CD36) (Supplementary Fig. 2). The fluorescent images and flow cytometry data showed that PAPC-L (PAPC:HSPC:Cholesterol = 3:5:2) are more specifically taken up by M2 than M1 macrophages in contrast to HSPC-L (Fig. 1e, f). On the contrary, PGPC-L showed a high uptake by both M1 and M2, as shown with fluorescent images and flow cytometry data, which might be attributed to the increase in size in cell culture medium (see Fig. 1d). We also confirmed that PAPC-L, at the lower ratio (PAPC:HSPC:Cholesterol = 2:6:2 and 1:7:2), also showed a higher uptake by M2 than M1 macrophages while no difference was seen with PGPC-L already at ratio (PGPC:HSPC:Cholesterol = 2:6:2) (Fig. 1g). As the M2/M1 uptake for PAPC-L was higher with PAPC-L (3:5:2), we selected this formulation for the targeting to M2 macrophages. Furthermore, we investigated whether PAPC-L do not induce toxicity in cells or self-activate macrophages. We found that PAPC-L did not self-activate macrophages towards M1 or M2 phenotypes (Supplementary Fig. 3a). Also, PAPC-L did not induce any toxicity in THP-1 derived macrophages, as shown with Alamar blue assay (Supplementary Fig. 3b).

**Fig. 2:**
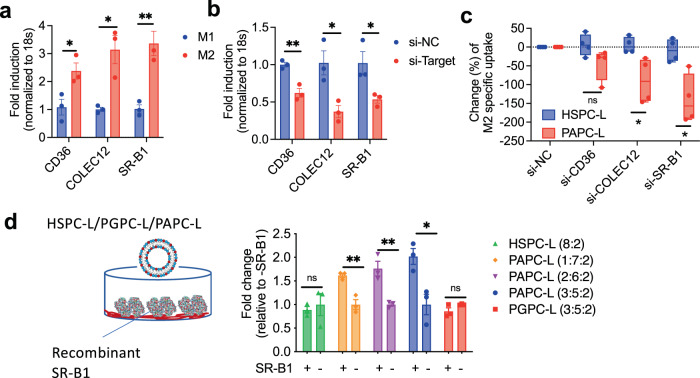
Role of scavenger receptors in uptake of M2-targeted nanoliposomes. **a** mRNA expression data showing the upregulation of M2 specific surface receptors CD36, COLEC12 and SR-B1 compared to M1 (left to right: *p = 0.032, *p = 0.013, **p = 0.0068). Data represents mean ± standard error of the mean (SEM) for three independent experiments. Statistical analysis was performed with multiple unpaired t-tests with correction for multiple comparisons using the Holm-Sidak’s method. **b** Knockdown of CD36, COLEC12 and SR-B1 using siRNA technique shows reduced gene expression levels of CD36, COLEC12 and SR-B1 in M2 macrophages (left to right: **p = 0.0049, *p = 0.022, *p = 0.040). Data represent mean ± SEM for three independent experiments. Statistical analysis was performed with Multiple unpaired t-tests with correction for multiple comparisons using the Holm-Sidak’s method. **c** Quantitation of flow cytometry analysis showing percentage change in M2-specific uptake after silencing of M2-specific surface receptors after incubation for 2 h (left to right: ns = non-significant, *p = 0.010, *p = 0.024). Data represent the box-and-whisker plot where every dot represents a data point from *n* = 4 independent experiments and the line in the box corresponds to the median. The boxes go from the upper to the lower quartiles, and the whiskers go from the minimum to the maximum value. Statistical analysis was performed with Multiple unpaired t-tests with correction for multiple comparisons using the Holm–Sidak’s method. **d** Binding data showing binding of HSPC-L (8:2), PGPC-L (3:5:2) and PAPC-L (1:7:2, 2:6:2, 3:5:2) to the human recombinant SR-B1 receptor (left to right: ns = non-significant, **p = 0.0058, **p = 0.0089, *p = 0.020). Data represent the mean ± SEM from three independent experiments. Unpaired two-sided Student’s *t*-test was used. Source data are provided as a Source Data file.

Earlier we have performed a gene array to examine differential phagocytic receptors and pathways in M1 and M2 macrophages and found that M2 macrophages overexpress a family of scavenger receptors CD36, COLEC12 and SR-B1 compared to M1^28^. We assumed that these receptors might play a role in the preferred uptake of PAPC-L by M2 macrophages. We confirmed the upregulation of these receptors in M2 macrophages compared to M1 (Fig. 2a) and further investigated the role of these pathways in the uptake of PAPC-L. We therefore knocked down these receptors using siRNA technique, as shown with qPCR in Fig. 2b and performed cell uptake in M2 macrophages using flow cytometry (Fig. 2c). We found that cells with reduced SR-B1 and COLEC12 expression had lower uptake of PAPC-L while there was no significant reduction in CD36 knockdown cells (Fig. 2c). Furthermore, to prove that PAPC-L indeed bind to SR-B1 receptor, we performed a binding study of PAPC-L, HSPC and PGPC-L with recombinant human SR-B1 receptor (Fig. 2d). Chimeric SR-B1 fused with Fc fragment of human IgG was displayed in the protein A coated plates and binding studies with DiI-labeled nanoliposomes were performed. We found that PAPC-L with increasing concentration of PAPC (10% to 30%) had an increase in the binding to the SR-B1 receptor, while HSPC-L and PGPC-L showed no increase in the binding signal (Fig. 2d). These data show that the PAPC-L are able to bind to the SR-B1 receptor and thereby taken up by M2 macrophages.

### Computational model revealing the tail-flipping mechanism of nanoliposomes

**Fig. 3:**
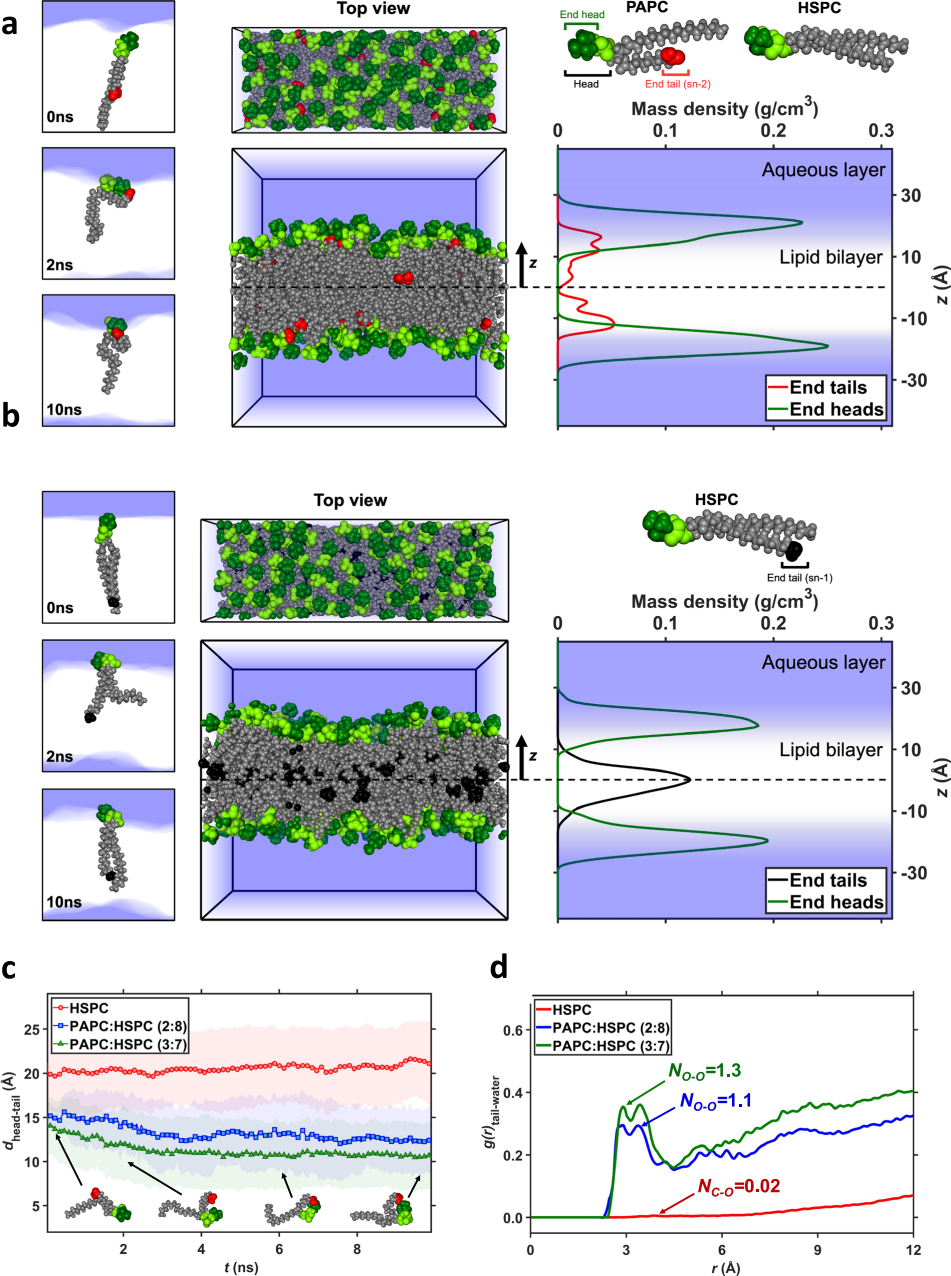
Computational simulation showing the “tail-flipping” phenomenon of PAPC in the bilayer of nanoliposomes. Top and side views of **a** PAPC:HSPC (3:7) and **b** pure HSPC bilayers at the end of 10 ns molecular dynamics (MD) simulations, along with the mass density profiles of the tops of all lipid heads and *sn-2* tail ends of PAPC or *sn-1* of HSPC. Atoms in head groups are highlighted in dark green, the oxygen atoms at the end of the *sn-2* tails are colored in red, and the carbon atoms at the end of the *sn-1* tails are colored in black. Their densities distributions along the bilayer normal are colored likewise. The blue zones depict aqueous regions. On the left side, snapshots of **a** a PAPC and **b** an HSPC lipid at the three times indicated, illustrating the markedly different locations of the tail ends of the two lipids. **c** The intra-molecular distance between the centers of mass of the head’s top and the *sn-2* tail’s end of PAPC in two mixed bilayers, and the *sn-1* tail’s end of HSPC in a pure bilayer, plotted against simulation time. Average distances are represented by markers, standard deviations by the shaded areas. The simulations started with straightened tails; the tails with carboxylated ends subsequently folded to bring the oxygen atoms near the head group, while the all-carbon tails remained elongated. The snapshots show conformations of a PAPC lipid at the times and distances indicated by the arrow heads. **d** Radial distribution functions g(r) of water oxygen atoms relative to the two oxygen (carbon) atoms at the end of the *sn-2* (*sn-1*) tail of PAPC (HSPC) in mixed (pure) bilayers, averaged over the last 1 ns of the simulation. Next to the curves are the average numbers N_O–O_ and N_C–O_ of contacts of per tail atom with oxygen atoms of water molecules in the first neighbor shell, i.e., contacts corresponding to the nearest-neighbor peak ending at 4.2 Å.

Earlier, it has been demonstrated that oxidized fatty acid tails of phospholipids in the cell membrane of apoptotic cells can protrude out and get recognized by macrophages via scavenger receptors resulting into the engulfment of apoptotic cells^34^. To understand whether the used carboxylated phospholipids (PAPC and PGPC) in our nanoliposomes biomimic the similar phenomenon as of oxidized fatty acid tails, we performed all-atom molecular dynamics simulations to visualize the distribution and behavior of PAPC and PGPC in bilayers. Four bilayer systems were considered, comprising two PAPC/HSPC systems with PAPC:HSPC (2:8 and 3:7), one PGPC/HSPC system with PGPC:HSPC (3:7), and a pure HSPC bilayer. Figure 3a shows side and top views of the PAPC:HSPC (3:7) bilayer at the end of the 10 ns molecular dynamic (MD) simulation, along with the mass density profiles, along the *z* direction normal to the bilayer, of the PAPC carboxylated tail ends and the tops of all head groups, averaged over the last 1 ns of the simulation (Supplementary Movie 1). The significant overlap of the distribution of carboxylated tail ends with the distribution of tops of heads indicates that these tail ends have moved away from their position in the mid-plane of the bilayer at the start of the simulation, to settle near the heads and in the vicinity of the water-bilayer interface at the end of the simulation. The snapshots in Fig. 3a of individual lipids at 0, 2 and 10 ns confirm this conformational transition. The short tail of HSPC, however, remain in the middle of the bilayer, as illustrated for HSPC in Fig. 3b and Supplementary Movie 2. by the non-overlapping mass density profiles of tail-ends and tops of heads. Supplementary Fig. 4a, b shows the distributions for the PAPC:HSPC (2:8) and PGPC:HSPC (3:7) systems, respectively (Supplementary Movies 3 and 4). In both cases, most carboxylated tail-ends atoms again reside near the water-bilayer interface.

The time evolution of the average intramolecular head-to-tail distances for two PAPC:HSPC bilayers and the pure HSPC system are presented in Fig. 3c. The head-to-tail distance of PAPC decreases rapidly, after starting the simulations with fully stretched tails, to adopt conformations with the carboxylated tail end anchored near the head. For the *sn-1* tails of HSPC, however, the head-to-tail distance remains at a high constant value. Both observations support the density profiles (Fig. 3a, b). Supplementary Fig. 4c shows that the head-to-tail distance of the short carboxylated *sn-2* tail of PGPC remains constant at around 9 Å over the simulation time, because the short tails flipped immediately after the start of the simulation.

To quantify the proximity of the tail ends to the water, Fig. 3d presents the radial distribution functions *g*(*r*) of PAPC/HSPC systems averaged over the last 1 ns. The radial distribution function denotes the ratio between the actual number of water molecules at a distance *r* versus the expectation value for this number for an atom homogeneously surrounded by water. Hence, the vanishing *g* at low *r* represents the excluded volume interaction between atoms, while for large distances *g* will approach unity. The carboxylated tail ends show a double peak starting at oxygen’s van der Waals radius of about 3 Å, corresponding to (a segment of) a first hydration shell, with little subsequent structure. This peak, ending at a distance of 4.2 Å, corresponds to each oxygen atom in PAPC on average being in direct contact with 1.1–1.3 oxygen atoms of water molecules. While the density profiles in Fig. 3a suggest that these tail ends are still largely shielded by the polar heads, this shows that the tail ends interact directly with the polar groups. A slightly higher coordination number of 1.5 was obtained for PGPC, as shown in Supplementary Fig. 4d. For reference, HSPC’s tail end interacts with 0.02 water molecules.

Overall, the all-atom simulation data of the bilayer models predict that the carboxylated *sn-2* tails of both PAPC and PGPC, similar to various oxidized phospholipids in cell membranes, are able to flip towards the aqueous layer rapidly^34^. Although the simulation shows that both PAPC and PGPC are able to display tail-flipping, the cell uptake experiments showed only M2-specific uptake by PAPC-L (see Fig. 1e–g) which was confirmed with the binding studies with the SR-B1 receptor (Fig. 2d). This means that a longer chain of terminal carboxylate, as in PAPC, is needed to make interactions with the receptors.

**Fig. 4:**
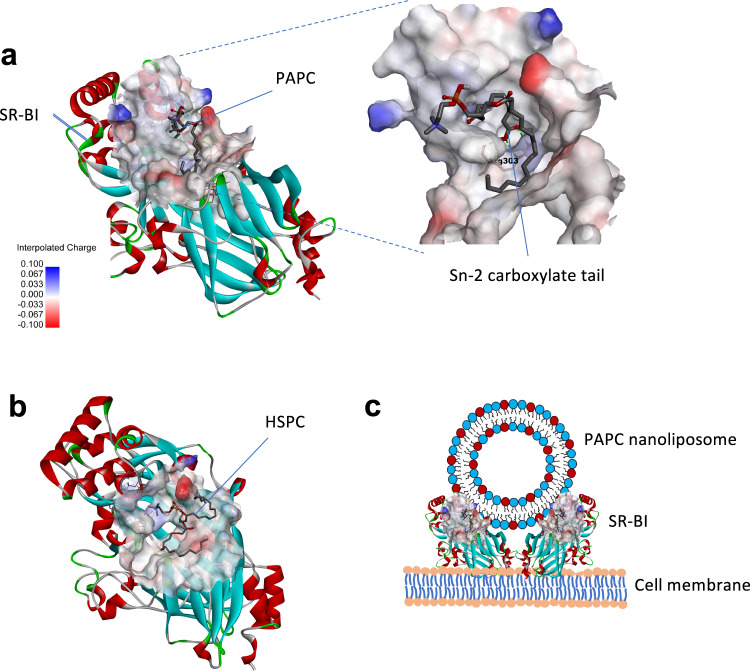
Molecular docking analysis for binding of PAPC and HSPC with SR-B1 receptor. **a** Docking image showing binding of PAPC with SR-B1 structure obtained using Autodock software. The SR-B1 structure show cationic sites (light blue color) which forms a pocket for the interaction with PAPC. The carboxylated *sn-2* tail forms hydrogen-bonds with Arg303 as shown in the enlarged image. **b** Image shows the interaction of HSPC with SR-B1 in which tails are not showing any contact with the receptor. **c** The illustration showing the potential interaction of PAPC-L with SR-B1 receptor on the cell membrane of the macrophage.

To better understand how PAPC incorporated into the lipid bilayer could molecularly interact with these receptors, we performed in silico molecular docking of PAPC with the abovementioned receptors using Autodock software (Fig. 4a). As the molecular 3D structure model for SR-B1 is not available, we first built up the 3D structure models for SR-B1 based on the lysosomal integral membrane protein-2 (LIMP-2, PDB: 4F7B) protein template alignment using Swiss-Model^35^. The sequences of both SR-B1 and LIMP-2 are shown in Supplementary Fig. 5a. The SR-B1 model showed 94% accuracy in the Ramachandran plot (Supplementary Fig. 5b). To determine the potential binding site, we performed a blind docking between PAPC and HSPC with SR-B1. We found PAPC clustered mostly at one site (shown in Fig. 4a) which was ranked highest with the lowest estimated binding energy of −2.97 kcal/mol (Run 1) compared to other runs (Supplementary Fig. 5c). Interestingly, a study by Conrad et al.^36^ showed that LIMP-2, an endomembrane receptor and a close family member of SR-B1, forms a dimer after lipid binding and recognized the phospholipid binding site which is in the same region as we have identified in this study. Also, earlier studies have revealed that SR-B1 forms a dimer to interact with lipids^37^, similar to LIMP-2. Furthermore, Conrad et al.^36^ also showed that phosphoserine-containing lipid vesicles interact with LIMP-2 due to the presence of cationic patches on the outer surface. We have found that SR-B1 possesses a positively charged surface (light blue color in Fig. 4a) which may allow for the interaction with PAPC-L. Also, we found a strong H-bond formation between the positively charged Arg303 and the carboxylated *sn-2* tail (Fig. 4a). In contrast, the docking results showed that HSPC, which lacks the carboxylated *sn-2* tail, had a poor interaction with SR-B1 (binding energy +3.7 kcal/mol, Supplementary Fig. 5c) and showed no tail interaction (Fig. 4b). These data suggest that the interaction between PAPC and SR-B1 is largely due to the flipped carboxylated *sn-2* tail and therefore PAPC nanoliposomes could bind to the receptor on the cell membrane, as illustrated in Fig. 4c. Also, our experimental data on the binding of nanoliposomes to isolated SR-B1 receptor (Fig. 2d) showing the specific interaction of only PAPC-L but not of HSPC-L confirms that the flipped *sn-2* tail is available on the PAPC nanoliposome surface to bind.

Furthermore, we also performed docking of PAPC and HSPC with COLEC12 (PDB: 2OX8) and CD36 (PDB: 5LGD) using Autodock software. We found that the PAPC had weaker binding to COLEC12 (binding energy −0.29 kcal/mol) and CD36 (+0.04 kcal/mol) compared to SR-B1 (−2.97 kcal/mol) (Supplementary Fig. 5c). HSPC had poor interactions with these receptors as indicated by the high binding energy (Supplementary Fig. 5c). The PDB files for the docking of PAPC and HSPC with SR-B1 are available in Supplementary Data 1. In line of these data, the experimental data in Fig. 2c showed that knocking down of SR-B1 resulted in the maximum reduction in the PAPC-L uptake by M2 macrophages compared to the knockdown of COLEC12 or CD36.

### PAPC nanoliposomes targets M2-type TAMs in vivo

**Fig. 5:**
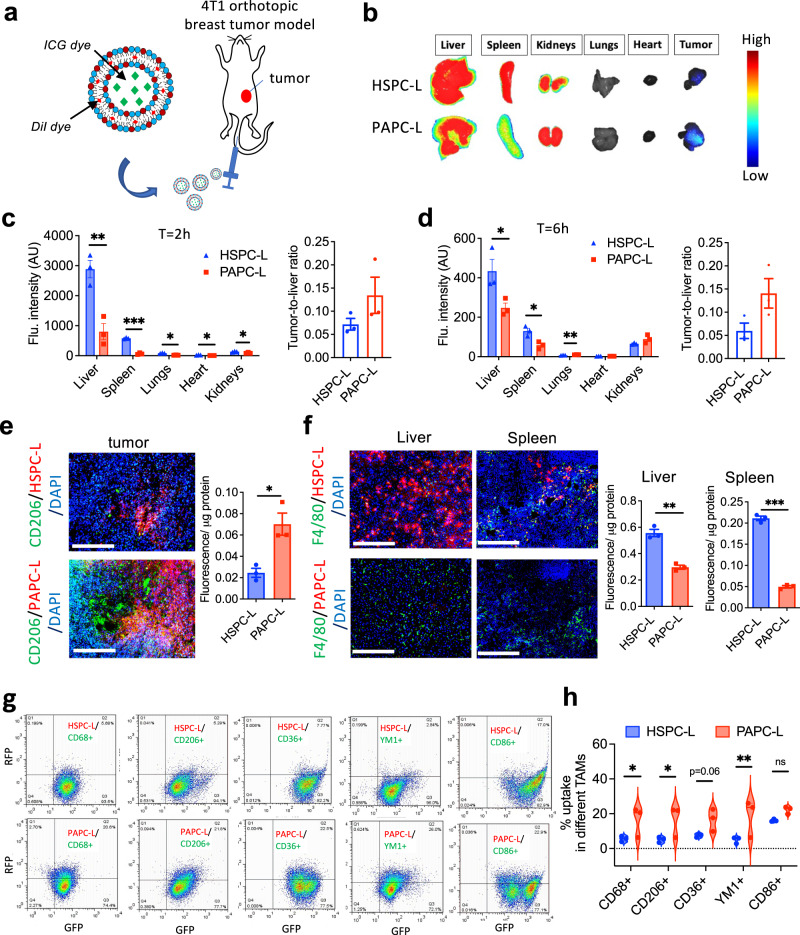
Tumor and organ distribution of PAPC nanoliposomes in vivo. **a** 1,1′-dioctadecyl-3,3,3′,3′-tetramethylindocarbocyanine (DiI) or indocyanine green (ICG) labeled PAPC-L were injected via tail vein into tumor bearing mice in 4T1 orthotopic breast tumor model. **b** Representative near-infrared images of different organs and tumor showing biodistribution of ICG-labeled HSPC-L and PAPC-L at *t* = 2 h. **c**, **d** Quantitative data of the fluorescent signal represent mean + standard error of the mean (SEM) from *n* = 3 mice per group at *t* = 2 h and *t* = 6 h (for (**c**) left to right: **p = 0.0062, ***p = 0.000019, *p = 0.0011, *p = 0.030, *p = 0.030; for (**d**), left to right: *p = 0.045, *p = 0.016, **p = 0.007). The statistical significance determined by Multiple unpaired *t*-tests with correction for multiple comparisons using the Holm–Sidak’s method. **e**, **f** Fluorescent microscopic images showing co-localization of DiI-HSPC-L/PAPC-L (red color) with CD206+ tumor-associated macrophages (TAM) (green color) within tumor tissues (e) or with F4/80+ macrophages (green color) in liver and spleen (f) (for (e), Tumor *p = 0.014; for (**f**), Liver **p = 0.0011, Spleen ***p = 0.000012). Nuclei, DAPI (blue color); Scale bar = 200 µm. Dil-labeled HSPC-L or PAPC-L were injected into tumor-bearing mice and tumors, liver and spleen were isolated at *t* = 1 h and homogenized to analyze total fluorescence per µg protein. Data represent mean + SEM from *n* = 3 mice per group. The statistical significance was determined by unpaired two-tailed student *t*-test. **g**, **h** Flow cytometric analysis showing histograms and quantitation of liposomal uptake into different populations of TAMs. Gated live cells stained with different TAM markers show the uptake of DiI-labeled HSPC-L or PAPC-L by TAMs. The bar graph shows the quantitative analysis of enhanced % uptake of PAPC-L by different TAMs (total TAMs: CD68+, M2/TAMs:CD206+, CD36+, YM1+ and M1/TAMs:CD86+) compared to HSPC-L (left to right: *p = 0.031, *p = 0.021, **p = 0.0084, ns non-significant). Data represent mean + SEM from *n* = 3 mice per group. The statistical significance determined by Multiple unpaired *t*-test for multiple comparisons considering single pooled variance. All statistical analyses were performed with Graphpad prism Ver. 9.3.1. Source data are provided as a Source Data file.

To investigate the overall biodistribution and tumor accumulation, we carried out different sets of experiments (Fig. 5a) and studied intra-tumoral distribution and macrophage uptake of DiI-/ Indocyanine green (ICG)-labeled PAPC-L in a triple-negative 4T1 orthotopic breast tumor mouse model. First, we showed that Dil containing HSPC-L and PAPC-L were stable and had no premature release of DiI when incubated in plasma at 37 °C until 6 h, while only a slight release at 24 h (Supplementary Fig. 6a). However, PGPC-L were found to be unstable and released the dye rapidly about 80% in 2 h and 100% in 24 h, therefore they were excluded from further in vivo studies.

We first set up an experiment to examine the overall biodistribution of PAPC-L and HSPC-L in different organs using ICG-labeled nanoliposomes to detect the distribution using an optical imager at a wavelength of 680 nm in the near-infrared (NIR) region. We first examined the stability of ICG-HSPC-L/PAPC-L in plasma and found them very stable until 6 h (Supplementary Fig. 6b). Then, after injection we tracked the animals until 6 hours but found no clear signal in intact animals to visualize different organs. But we isolated tumor and organs at 2 h and 6 h and scanned them with the animal imager (Fig. 5b, Supplementary Fig. 6c). The quantitation of the NIR signal showed that at *t* = 2 h, both ICG-labeled HSPC-L and PAPC-L largely accumulated in liver but PAPC-L had a significantly lower accumulation in liver as well as in spleen, lungs, heart and kidneys compared to HSPC-L (Fig. 5c). At *t* = 6 h, the total signal was reduced due to clearance of the nanoparticles from the body. Yet there was a lower signal of ICG-PAPC-L in liver and spleen compared to ICG-HSPC-L (Fig. 5d). Importantly, at both timepoints, the tumor-to-liver ratio was higher in ICG-PAPC-L treated mice compared to ICG-HSPC-L (Fig. 5c, d). To quantitate and visualize the organ and tumor distribution, we set up another experiment at an earlier timepoint. We injected DiI-labeled nanoliposomes and isolated tumor and organs at *t* = 1 h and examined fluorescence in tissue homogenates and performed double immunofluorescent staining to co-localize with CD206+ TAMs and F4/80+ macrophages in healthy organs. Remarkably, we found that PAPC-L had a significantly higher tumor accumulation compared to HSPC-L, which was associated with CD206+ M2 macrophages, as shown with immunofluorescent staining for CD206 (green) and nanoliposomes (red) and fluorescence analysis in tissue homogenates (Fig. 5e). As most nanoparticles are sequestered by liver and spleen as a part of reticuloendothelial system, we found that HSPC-L were strongly accumulated into liver and spleen in F4/80+ macrophages (Kupffer cells in liver and splenic macrophages), while PAPC-L showed a much lower accumulation in these organs (Fig. 5f). Furthermore, to examine the distribution of DiI-labeled nanoliposomes in different TAM populations, we performed a third experiment in which we isolated tumors after the injection of either DiI-labeled HSPC-L or PAPC-L. To demonstrate that PAPC-L have low accumulation in M1 type, we first performed co-localization studies with MHC-II, a M1 marker. We found that there were very few MHC-II positive cells in these tumors (Supplementary Fig. 6d). Importantly, PAPC-L (red color) accumulation had hardly any colocalization with MHC-II positive (green color) cells. Overall, there was much higher accumulation of PAPC-L in tumors compared to HSPC-L (Supplementary Fig. 6e). We performed flow cytometry to colocalize the accumulation of nanoliposomes with M1 or M2 TAM biomarkers as follows: total TAMs: CD68+; M1 TAMs: CD86+; M2 TAMs: CD206+, CD36+, YM1+ (see gating strategy in Supplementary Fig. 7). The flow cytometry data showed that total TAMs and M2 TAMs had a higher uptake of PAPC-L compared to HSPC-L, as shown in Fig. 5g, h. Interestingly, we also observed an uptake of PAPC-L by CD86+ M1 TAMs, however, found that CD86 is also expressed by a M2 subset M2b^38^, which could be a reason for this higher uptake. Altogether, these data demonstrate that PAPC-L, in contrast of HSPC-L, are able to target M2 TAMs within the tumor, while showing lesser accumulation into healthy organs.

### Altering TAMs using PAPC nanoliposomes

To evaluate the potential of our technology to alter M2-TAMs, we made a strategy to either (i) inhibit M2 differentiation by targeting a STAT6 inhibitor, (ii) deplete TAMs by targeting zoledronic acid, or (iii) train TAMs towards anti-tumoral M1 phenotype by targeting muramyl tripeptide (MTP). We then examined the direct effect on these TAM alterations on the tumor growth and pre-metastatic niche formation in lungs in orthotopic 4T1 breast tumor model.

**Fig. 6:**
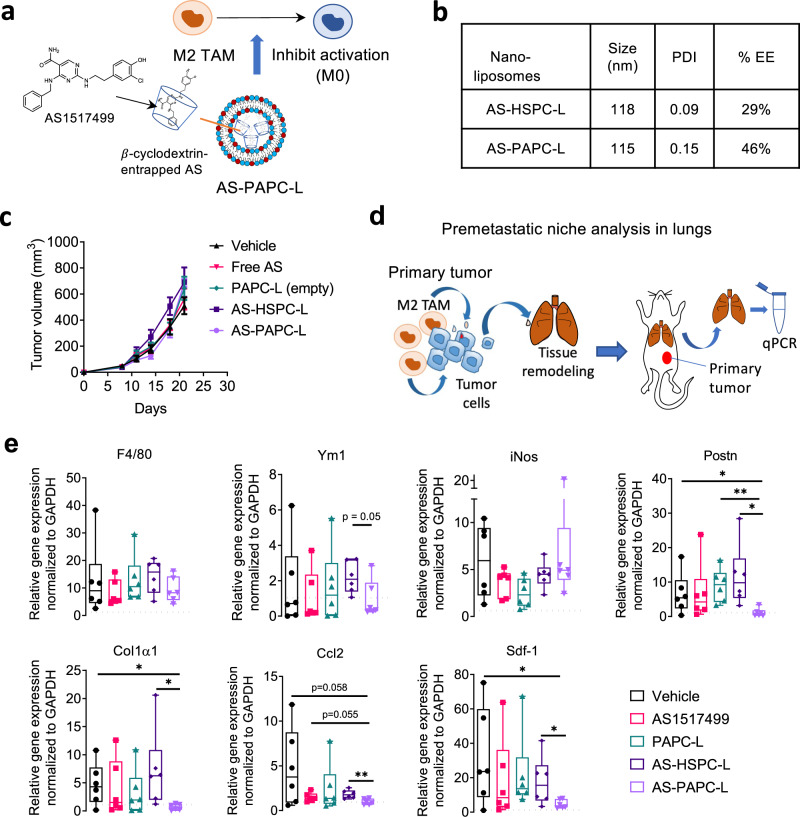
Targeting STAT6 inhibitor AS1517499 (AS) using PAPC-L. **a** A diagram showing AS encapsulated PAPC nanoliposomes (AS-PAPC-L) inhibiting the activation of M2 TAMs. **b** Physicochemical properties of AS-HSPC-L and AS-PAPC-L and encapsulation efficiency (%EE). **c** Tumor growth curves showing that none of the treatments including empty PAPC-L, free AS, AS-HSPC-L and AS-PAPC-L on the tumor growth of orthotopic 4T1 breast tumor model in mice. Free AS, AS-HSPC-L and AS-PAPC-L (dose equivalent to 8 mg/kg AS, 100 µl injection volume) were injected (free AS, i.p.; nanoliposomes, i.v.) twice a week when tumors became ±100 mm^3^. Mean + standard error of the mean (SEM), *N* = 6 mice per group. **d** Diagram illustrating the premetastatic niche formation in lungs. M2 TAMs secrete cytokines which activate tumor cells and in response of that tumor cells secrete factors including cytokines, extracellular vesicles which form metastatic niche in lungs. Lungs from the tumor-bearing mice were isolated and analyzed with quantitative polymerase chain reaction (qPCR). **e** Graphs showing quantitative real-time PCR results for the effect of AS-HSPC-L and AS-PAPC-L on the markers for pre-metastatic niche in lungs. *N* = 6 mice per group (left to right, Postn: *p = 0.049, **p = 0.0032, *p = 0.017; Col1α1: *p = 0.025, *p = 0.041; Ccl2 **p = 0.0046; Sdf-1: *p = 0.041, *p = 0.049. Data represent the box-and-whisker plot where every dot represents a data point and the line in the box corresponds to the median. The boxes go from the upper to the lower quartiles, and the whiskers go from the minimum to the maximum value. Statistical analysis was performed with unpaired two-tailed Student’s *t*-test. Source data are provided as a Source Data file.

Targeting Stat6 inhibitor AS1517499 to inhibit M2 activation: Previously, we have studied the role of STAT6 pathway, the IL-4/IL-13 mediated downstream pathway, in inhibition of M2 differentiation and M2-driven tumor cell migration in breast tumor model using the STAT6 inhibitor AS1517499 (AS)^22^. In this study, we encapsulated AS into HSPC-L or PAPC-L by first creating entrapping AS into hydroxypropyl methyl β-cyclodextrin and then subsequently into nanoliposomes (Fig. 6a). Based on %EE, 0.14 mol AS per lipid mol was encapsulated in AS-HSPC-L, while 0.23 mol AS per lipid mol in AS-PAPC-L.

The physicochemical properties and drug encapsulation of AS-HSPC-L and AS-PAPC-L are shown in Fig. 6b. In vitro, we confirmed that the encapsulation of AS in nanoliposomes had no toxic effect on cells and did not induce M1 phenotype (Supplementary Fig. 8a, b) but inhibited M2 marker Mrc-1 and Arg-1 (Supplementary Fig. 8c). In vivo, the treatment with free AS, AS-HSPC-L or AS-PAPC-L (equivalent to 8 mg/kg AS) did not show any inhibitory effect on the tumor growth (Fig. 6c). Previously, to study the role of STAT6 pathway in vivo, we used 20 mg/kg dose of AS in which we observed a slight inhibition of the tumor growth and a major reduction in the lung premetastatic niche formation^22^. In this study, we therefore investigated the effect of AS-PAPC-L on the pre-metastatic niche in lungs using qPCR as shown in Fig. 6d. We used several pre-metastatic niche markers, including macrophage markers (all: F4/80; M2: Ym1; M1: iNos), secretory factors (Sdf-1, Postn, Ccl2), and ECM (Col1α1). We found that lungs from the tumor-bearing mice had an increased expression of all these factors compared to healthy lungs (see dotted lines in Fig. 6e). Interestingly, AS-PAPC-L specifically inhibited the gene markers of pre-metastatic niche in contrast to free AS, empty PAPC-L, and AS-HSPC-L, as shown in Fig. 6e. In particular, the markers induced during the metastatic niche formation such as ECM (Postn, Col1α1) and cytokines responsible for inducing metastasis (Ccl2, Sdf-1α) were inhibited. These data indicated that targeting of AS using PAPC-L inhibits M2-TAM driven premetastatic niche formation. Earlier we have demonstrated that the inhibition of STAT6 in M2 macrophages only hampered the M2-induced tumor cell migration as shown in in vitro cultures using conditioned media and in vivo in 4T1 tumor model^22^. This explains why targeting of STAT6 inhibitor as AS-PAPC-L only showed an effect on the premetastatic niche formation.

**Fig. 7:**
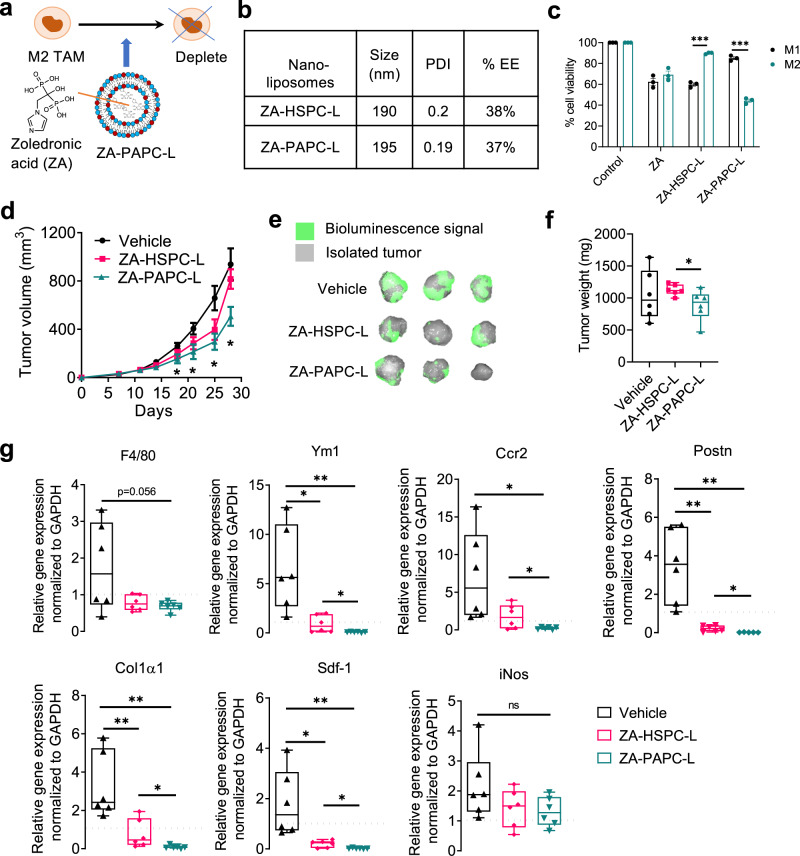
Targeting zoledronic acid (ZA) using PAPC-L. **a** A diagram showing ZA encapsulated PAPC nanoliposomes (ZA-PAPC-L) depleting M2 TAMs. **b** Physicochemical properties of AS-HSPC-L and AS-PAPC-L and encapsulation efficiency (%EE). **c** % cell viability of THP-1 differentiated M1 and M2 macrophages 24 h after the treatment of free ZA, ZA-HSPC-L or ZA-PAPC-L (left to right: ***p = 0.000043, ***p = 0.000049). Data represents mean ± standard error of the mean (SEM) for three independent experiments. Statistical analysis was performed with Multiple unpaired *t*-tests with correction for multiple comparisons using the Holm–Sidak’s method. **d** Tumor growth curves in orthotopic 4T1 breast tumor model in mice. Both ZA-HSPC-L and ZA-PAPC-L were injected with dose equivalent to 0.5 mg/kg ZA, 100 µl injection volume, i.v., twice a week, when tumors became ±100 mm^3^. Before sacrifice, mice were injected with 2.5 mg of D-luciferin and were imaged after 10 min to detect bioluminescence signal using Pearl Trilogy imager (LICOR) (left to right: *p = 0.049, *p = 0.034, *p = 0.014, *p = 0.018 between Vehicle and ZA-PAPC-L). Mean + SEM, *N* = 6 mice per group, Statistical analysis was performed with unpaired two-tailed Student’s *t*-test. **e** Representative images showing the isolated tumors with bioluminescence signal (green). **f** Graphs showing the tumor weight of the isolated tumors at the end of the experiment. *N* = 6 mice per group (*p = 0.043). Statistical analysis was performed with unpaired two-tailed Student’s *t*-test. **g** Box graphs showing quantitative real-time PCR results showing the effect of ZA-HSPC-L and ZA-PAPC-L on different markers for pre-metastatic niche in lungs. *N* = 6 mice per group (left to right, Ym1: *p = 0.010, **p = 0.0043, *p = 0.044; Ccr2: *p = 0.018, *p = 0.042; Postn: **p = 0.0020, **p = 0.0031, *p = 0.021; Col1α1: **p = 0.0088, **p = 0.0011, *p = 0.048; Sdf-1: *p = 0.014, **p = 0.0079, *p = 0.012; iNos: ns = non-significant). Data in (**f** and **g**) represent the box-and-whisker plot in which every dot represents a data point and the line in the box as median; the box shows the upper to the lower quartiles; the whiskers show the minimum to the maximum value. Statistical analysis was performed with unpaired two-tailed Student’s t-test. Source data are provided as a Source Data file.

Targeting zoledronic acid to deplete TAMs: To deplete macrophages, we incorporated a well-known apoptosis-inducing compound zoledronic acid (ZA) into HSPC-L and PAPC-L (Fig. 7a). This resulted into ZA-HSPC-L (∅ = 190 nm) and ZA-PAPC-L (∅ = 195 nm) formulations (Fig. 7b). Based on the %EE, 0.27 mol ZA was encapsulated in 1 mol total lipid in both ZA-HSPC-L and ZA-PAPC-L.

In vitro, ZA-PAPC-L reduced cell viability specifically in human THP-1 differentiated M2 macrophages compared to M1 macrophages (Fig. 7c). In contrast, free ZA showed inhibitory effects in both M1 and M2 equally and ZA-HSPC-L showed reduced viability in M1 but not in M2. In vivo, ZA-PAPC-L showed a significant reduction of the tumor growth in 4T1 triple-negative breast tumor model (Fig. 7d), which can be observed at the isolated tumors and end tumor weights (Fig. 7e, f). Similar to AS-PAPC-L study, we also investigated the effect of ZA-PAPC-L on the pre-metastatic niche markers (Fig. 7g). Remarkably, treatment with ZA-PAPC-L strongly reduced the markers of total macrophage (F4/80), M2 macrophages (Ym1) but not of M1 macrophages (iNOS). Furthermore, other pre-metastatic niche markers were also inhibited significantly with ZA-PAPC-L compared to the vehicle group (Fig. 7g). In contrast to the results with AS-PAPC-L, we found that the treatment with ZA-PAPC-L inhibited most markers much stronger and below the healthy lung levels, which might attribute to the induced toxicity in M2 macrophages by ZA-PAPC-L. Although we expect that these effects are due to the tumor inhibitory effects on the primary tumor, there is a plausibility that ZA-PAPC-L cause direct effects in lungs, leading to lower gene levels compared to healthy lungs. In case of both AS-PAPC-L and ZA-PAPC-L, we observed no apparent toxicity in animals as examined with the no change in organ weights (Supplementary Fig. 9a, b). Furthermore, as shown in Supplementary Fig. 9a, the treatment with AS-PAPC-L ameliorated the reduced spleen weight which was seen in tumor-bearing mice compared to healthy animals (dotted line).

Furthermore, we also examined the anti-tumoral effects of ZA-PAPC-L and ZA-HSPC-L in another tumor model CT26 colon carcinoma in balb/c mice to demonstrate the benefit of the targeting. We found that ZA-PAPC-L showed a reduction of the tumor growth compared to the control vehicle and ZA-HSPC-L groups (Supplementary Fig. 10a, b). Unfortunately, free ZA was found to be toxic and therefore this group had to be terminated. These data indicate that delivery of ZA using PAPC-L was safe and more effective. Furthermore, we examined lungs for the effect on the metastatic niche markers (Supplementary Fig. 10c) and found that macrophage marker F4/80 was significantly reduced by ZA-PAPC-L indicating the direct inhibitory effect on the macrophages. Interestingly, there was a significant increase in iNOS expression by ZA-PAPC-L but not by ZA-HSPC-L, suggesting that ZA-PAPC-L induced the re-polarization to M1 type. In case of other markers, there were also inhibitory trends which imply that ZA-PAPC-L has an inhibitory effect on the metastatic niche formation in this model.

**Fig. 8:**
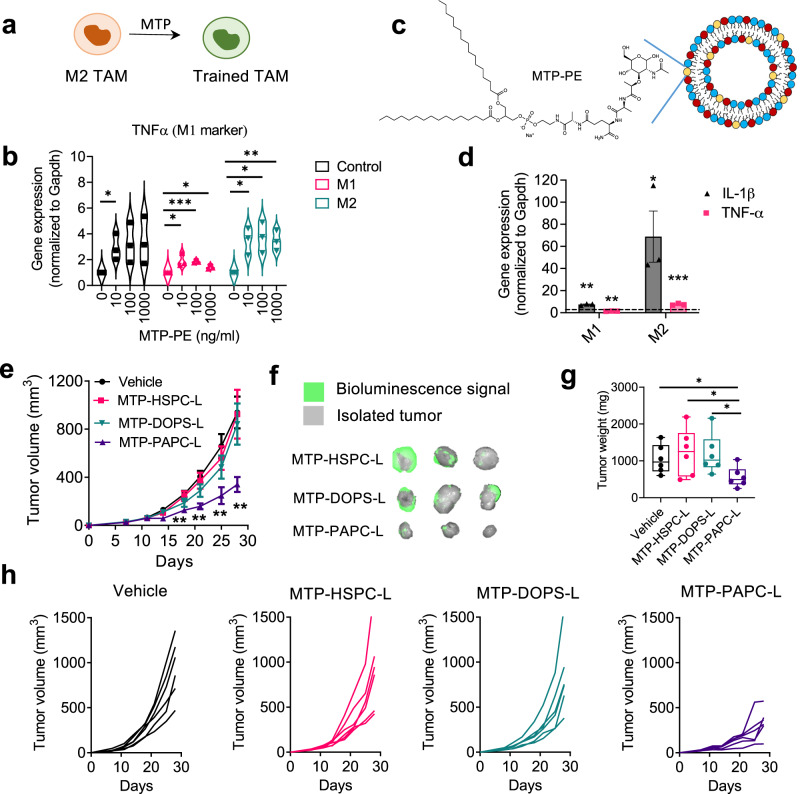
Training TAMs by targeting muramyl tripeptide (MTP)-PE using PAPC-L. **a** A diagram showing the role of MTP in training M2 TAMs into M1 macrophages. **b** Gene expression data showing that treatment of RAW264.7 macrophages with MTP-PE leads to the induction of TNF-α as well as of IL-1β (see Supplementary Fig. 11)(left to right, Control: *p = 0.030; M1: *p = 0.032, ***p = 0.00037, *p = 0.010; M2: *p = 0.0150, *p = 0.016, **p = 0.0058). Data represented as Violin plot for three independent experiments. Statistical analysis was performed with Multiple unpaired t-tests with correction for multiple comparisons using the Holm–Sidak’s method. **c** MTP-encapsulated PAPC nanoliposomes (MTP-PAPC-L) incorporating MTP-PE in the lipid bilayer. **d** Gene expression data showing that treatment with MTP-PAPC-L induced the expression of M1 markers (IL-1β and TNF-α) in M2 macrophages to a much higher extent than in M1 macrophages (left to right: **p = 0.00013, **p = 0.0099, *p = 0.042, ***p = 0.00019). Data represents mean ± standard error of the mean (SEM) for three independent experiments. Statistical analysis was performed with Multiple unpaired t-tests with correction for multiple comparisons using the Holm-Sidak’s method. **e** Tumor growth curves showing the effect on the tumor growth in 4T1 breast tumor model in mice. Dose of MTP-PE equivalent to 1 mg/kg/mouse, 100 µl intravenous, twice a week. (left to right: **p = 0.0027, **p = 0.0011, **p = 0.0075, **p = 0.0022 between Vehicle and MTP-PAPC-L). Mean + SEM., *N* = 6 mice per group, Statistical analysis was performed with unpaired two-tailed Student’s *t*-test. **f** Representative images showing the isolated tumors with bioluminescence signal (green). The vehicle group was the same as shown in Fig. 7 as experiments were run in parallel. **g** Box graph showing the tumor weight of the isolated tumors at the end of the experiment (left to right: *p = 0.030, *p = 0.039, *p = 0.029). *N* = 6 mice/group. Data represent the box-and-whisker plot in which every dot represents a data point and the line in the box as median; the box shows the upper to the lower quartiles; the whiskers show the minimum to the maximum value. Statistical differences were determined by unpaired two-tailed Student’s *t*-test. **h** Curves showing the tumor growth of individual tumor in different treatment groups. Source data are provided as a Source Data file.

Targeting muramyl tripeptide (MTP) to train TAMs: As the third strategy, we aimed to train TAMs to attain M1-like phenotype, the anti-tumoral phenotype, using MTP-PE (Fig. 8a). We first examined the effect of MTP-PE on the differentiation of mouse (RAW264.7) and human (THP-1) macrophages in M0 and M1 and M2 differentiation media. A successful differentiation to M1 and M2 was confirmed in both RAW264.7 and THP-1 cells, as shown with specific markers (Supplementary Fig. 2 and Supplementary Fig. 11a). Interestingly, MTP-PE stimulated both M1 and M2 macrophages more toward M1 type, as shown with the induced typical M1 markers (IL-1β and TNF-α) (Fig. 8b, Supplementary Fig. 11B, c). Earlier studies have shown that liposomal MTP-PE activated systemic macrophages into tumoricidal macrophages which could reduce lung metastasis in fibrosarcoma mouse model^39^. However, macrophage subtypes were not known during these studies. We then incorporated MTP-PE into the lipid bilayer of HSPC-L and PAPC-L resulting into MTP-HSPC-L (∅ = 123 nm; PDI 0.09) and MTP-PAPC-L (∅ = 173 nm; PDI 0.2) (Fig. 8c).

As the incorporation of MTP-PE into the lipid bilayer may affect the uptake behavior, we examined the uptake of MTP-PAPC-L into M1 and M2 macrophages and found that MTP-PAPC-L had still a much higher uptake by M2 than M1, as shown with fluorescence microscopy (Supplementary Fig. 12a). Surprisingly, the flow cytometry data showed that MTP-HSPC-L also had a higher uptake by M2 macrophages, but this was much lower compared to MTP-PAPC-L (Supplementary Fig. 12b). Furthermore, the treatment with MTP-PAPC-L induced the expression of M1 markers (IL-1β and TNF-α) in M2 macrophages to a much higher extent than in M1 macrophages (Fig. 8d), indicating the training of M2 to attain M1 phenotype.

**Fig. 9:**
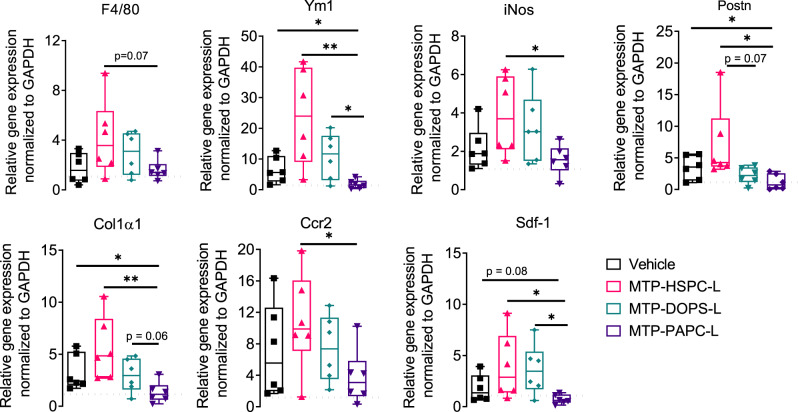
Effect of MTP-incorporated nanoliposomes on pre-metastatic niche markers. Box graphs showing quantitative real-time PCR results showing the effect of MTP-HSPC-L, MTP-DOPS and MTP-PAPC-L on different markers for pre-metastatic niche in lungs. *N* = 6 mice per group (left to right, Ym1: *p = 0.028, **p = 0.0065, *p = 0.015; iNos *p = 0.027; Postn: *p = 0.033, *p = 0.036; Col1α1: *p = 0.038, **p = 0.0088; Ccr2: *p = 0.038; Sdf-1: *p = 0.038, *p = 0.016). Data represent the box-and-whisker plots where every dot represents a data point and the line in the box corresponds to the median. The boxes go from the upper to the lower quartiles, and the whiskers go from the minimum to the maximum value. Statistical analysis was performed with unpaired two-tailed Student’s *t*-test. Source data are provided as a Source Data file.

Intriguingly, treatment with MTP-PAPC-L strongly attenuated the tumor progression by 70% in orthotopic 4T1 breast tumor model (Fig. 8e). In contrast, MTP-HSPC-L and other nanoliposomes MTP-DOPS-L (lipid composition = POPC: DOPS: MTP-PE = 6.83: 3: 0.16) containing DOPS (1,2-dioleoyl-sn-glycero-3-phospho-L-serine). DOPS has been earlier proposed to induce macrophage uptake^39^. In our study, however, it did not show any reduction in the tumor growth (Fig. 8e), which might be due to non-specific and inefficient targeting. These effects were also apparent from the isolated tumors and tumor weight and at individual tumor growth (Fig. 8f–h). Furthermore, we analyzed the lungs to examine the effect on the pre-metastatic niche formation. We found that MTP-PAPC-L clearly reduced the infiltration of monocytes (F4/80) and activation to M2 type (Ym1) as well as the secretion of pro-tumorigenic and pro-migratory cytokines (Sdf-1α, Postn) and ECM remodeling (Col-1α1) (Fig. 9). We surprisingly found that iNOS expression is also reduced to the normal level, which indicates that the inhibitory effects of MTP-PAPC-L on pre-metastasis niche formation is likely due to its inhibitory effects on the primary tumor. Furthermore, we observed no toxic effects on macrophage markers or other markers similar to ZA-targeted nanoliposomes, indicating that MTP-PAPC-L treatment was highly effective and non-toxic. Furthermore, we also found no side effect on the organ weights and the body weight (Supplementary Fig. 13a, b).

## Discussion

Alternatively-activated M2-like macrophages have been regarded as key target cells in malignant diseases as well as in nonmalignant diseases such as asthma^33^. This study presents novel “tail-flipping” nanoliposomes which target M2-like macrophages via the biomimicry of anionic and oxidized phospholipids on apoptotic cells which are being recognized by macrophages as a natural clearance mechanism. The *sn-2* terminal carboxylated phospholipid PAPC is able to flip back its charged hydrophilic tail towards the external aqueous phase upon incorporation into the lipid bilayer and thereby interacts with M2-upregulated SR-B1 receptor. Similar interactions have been observed between other phospholipids or cholesterol with endomembrane scavenger receptors such as LIMP-2, a close member of the SR-B1 receptor family^36^. Co-crystallization studies showed that the LIMP-2 receptor forms a tunnel to transport phospholipids intracellularly. Our molecular docking studies predict that SR-B1 interacts with negatively charged PAPC lipid in a similar way (Fig. 4a) and help engulf nanoliposomes, which need further analysis using co-crystallization of PAPC with SR-B1, followed by structure analysis with x-ray crystallography.

In vivo, PAPC-L showed a higher tumor accumulation and more importantly specific targeting to M2-subpopulation of TAMs was seen in an orthotopic breast tumor model compared to untargeted nanoliposomes. Sequestration of nanoparticles by the monocytic phagocytic system such as Kupffer cells in liver and immune cells in spleen is a major problem for nanomedicine to reach targets in the tumor microenvironment^40^. A lower accumulation of PAPC-L by liver and spleen compared to HSPC-L signifies the benefit of our targeting system.

To convincingly demonstrate the effectiveness of our targeting approach, three different therapeutic agents were delivered by our nanoliposome system to M2-TAMs to alter their functionality either by inhibiting the STAT6 pathway using AS, inducing their depletion using ZA or training them using MTP. As shown earlier, inhibition of STAT6 with AS led to inhibition of macrophage-induced tumor cell migration^22^. In this study, the inhibition of pre-metastatic niche with the AS-PAPC-L treatment indicates the inhibition of the paracrine effect of TAMs which is in the line of the latter study, while a direct effect on macrophages in the lung niche remains to be investigated. In contrast of AS-PAPC-L, ZA-PAPC-L reduced the F4/80 expression below the normal lung levels (Fig. 7g vs. Fig. 6e) which suggests that there might be a direct effect on lung macrophages. Among all approaches, we found that targeting of MTP to M2-TAMs was the most successful one showing approximately 70% reduction in the tumor growth as well as inhibition of pre-metastatic niche formation in lungs.

Furthermore, the advantage of these nanoliposomes is that they are easy to prepare with high consistency and reproducibility, which makes them suitable to scale-up. Since a phospholipid acts as a targeting ligand in PAPC-L rather than an antibody or a peptide, no surface chemistry is needed to target nanoparticles. This is a great advantage over other approaches which depend on chemical surface functionalization of nanoparticles, leading to extra steps of chemical synthesis and purification as well as add batch-to-batch variability. In conclusion, our nanoliposome system represents an effective and versatile tool system to target and alter M2-TAMs, which can be exploited for developing novel immunotherapies against cancer.

## Methods

### Preparation of nanoliposomes

Nanoliposomes are prepared using the ethanol injection technique as explained earlier^41^. Briefly, hydrogenated soy phosphatidylcholine (HSPC, Lipoid GMBH, Germany), oxidized lipids 1-palmitoyl-2-azelaoyl-sn-glycero-3-phosphocholine (PAPC, Cayman chemicals, Ann Arbor, MI) or 1-palmitoyl-2-glutaryl phosphatidylcholine (PGPC, Cayman chemicals), cholesterol (Sigma Aldrich) and the lipophilic fluorescent label DiI (Sigma Aldrich, St Louis, MO) at a molar ratio of 0.1:9.9 (mol: mol) of total lipid were dissolved in ethanol and heated at 70 °C. The heated lipid solution was injected into PBS to create a crude liposomal formulation with a concentration of 10 mM total lipid. The ethanol to PBS ratio was always 1:10. The crude liposomes were extruded through 400 nm, 200 nm and 100 nm polycarbonate filters using the Avestin Liposofast extruder. Once extruded the liposomes were further purified by passing through a PD10 column (GE Healthcare) by eluting it at 1000 g for 2 min. The purified liposomes were always stored at 4 °C till use.

### Drug encapsulation into nanoliposomes

Preparation of AS-loaded nanoliposomes: Nanoliposomes were prepared using the ethanol injection method as previously described. AS was dissolved in DMSO at 50 mM solution, which was subsequently dissolved in 10% 2-hydroxypropyl-β-cyclodextrin solution (1:9 v/v) forming a complex of AS-cyclodextrin solution. Different lipids (PAPC: HSPC: cholesterol) were dissolved in 100% ethanol in the molar ratio of 0.3:0.5:0.2 (mol: mol: mol). The AS-cyclodextrin solution (1000 µl) was injected with lipids dissolved in ethanol (110 µl). The nanoliposomes were repeatedly extruded and purified as explained above. To calculate the % encapsulation efficiency (%EE), nanoliposomes were diluted 1:10 with 100% ethanol, sonicated for 30 min and measured using ND-1000 spectrophotometer (NanoDrop, Thermo Fisher Scientific) at 261 nm and 306 nm. A calibration curve for AS1517499 was plotted before measuring the liposomes.

Preparation of ZA-loaded nanoliposomes: Nanoliposomes were prepared using thin film hydration method. Different lipids (PAPC: HSPC: cholesterol) were collected in a tube in the molar ratio of 0.3:0.5:0.2 (mol: mol: mol). The solvent was evaporated under the flow of N2 gas at 70 °C and redissolved in 1 ml 100% ethanol. The lipid solution was pipetted in a round bottom flask and a thin lipid film was formed, to which a pre-heated solution of ZA dissolved in PBS (5 mg/ml) was added. Once rehydrated the crude liposomes were repeatedly extruded and purified using the PD10 column as described above. The %EE was calculated using ND-1000 spectrophotometer (NanoDrop, Thermo Fisher Scientific) at 228 nm.

Preparation of MTP-PE-loaded nanoliposomes: Nanoliposomes were prepared using the thin film hydration method. All the lipids and MTP-PE (HSPC: PAPC: cholesterol: MTP-PE) were collected in an eppendorf in the molar ratios of 0.48:0.3:0.2:0.016 (mol: mol: mol: mol). The solvents were evaporated under the flow of N2 gas at 70 °C. Fresh 1 ml of 100% ethanol was added to the dried film of lipids to re-dissolve them. The same procedure of thin-film layer and extrusion was followed as explained for ZA nanoliposomes. The incorporation of MTP-PE in the lipid bilayer was analyzed using the Ultimate® 3000 uHPLC with Charged Aerosol Detector, CAD (Dionex™ Corona™; Thermo Scientific) using C18 UPLC column. The mobile phase consists of 80:20 Methanol: H2O with 0.05% TEA (A), 100% Methanol with 0.05% TEA (B) and a constant flow rate of 0.3 ml/min. The data was analyzed using Chromeleon® Chromatography data system. Before analysis, nanoliposomes were diluted 1:10 in ethanol and sonicated for 30 minutes.

### Physicochemical characterization

Liposomal size (in PBS) and zeta potential (in 0.1 mM KCl) were measured using Zetasizer Nano ZS (Malvern, Malvern, UK). The lipid content in the nanoliposomes was analyzed using the Ultimate® 3000 uHPLC with Charged Aerosol Detector, CAD (Dionex™ Corona™; Thermo Scientific) using C18 UPLC column. The mobile phase consists of methanol with 0.05% TFA (A), 80% methanol with 0.05% TFA (B) and a constant flow rate of 0.2 ml/min. The data was analyzed using Chromeleon® Chromatography data system. Before analysis, nanoliposomes were evaporated under N_2_ gas at 70 °C, then dissolved in methanol to disrupt the formulation as described above.

### Binding of liposomes to SR-B1 receptor

Liposomes were prepared in different ratios of carboxylated lipid (PAPC or PGPC) with lipophilic DiI dye. Protein A-coated 96 well plate (ThermoFisher Scientific) was washed with PBS for 1 minute over a shaking plate at 300 rpm. A hundred microliters of 1 μg/ml human recombinant protein SR-B1 (R&D systems) dissolved in PBS was added to the protein A-coated well plate. The plate was placed on a shaker at 300 rpm for 1 h. The plate was washed with PBS to eliminate the unattached protein in the wells. The SR-B1 protein coating was blocked with 2% BSA for 1 h. The excess blocking was washed with PBS and 100 μM liposomes (equal fluorescence) were added to the plate and kept for 1 h on a shaker at 300 rpm. The unattached liposomes were washed with PBS. The well plate was washed with 100 μl dry DMSO, to disrupt the liposomes and detach the protein from the well plate. Of the 100 μl, 90 μl of DMSO was collected in a flat bottom black 96-well plate. The fluorescence was measured from 505 nm to 567 nm with the TECAN plate reader with optimal gain.

### Stability studies

Nanoliposomes loaded with lipophilic dye DiI or hydrophilic dye ICG were mixed mouse-derived plasma in a ratio of 1:1. The liposome-plasma mixture was incubated for different time points (0, 2, 6, and/or 24 h) at 37 °C. The release of dye from the liposomes was detected by the loss of fluorescence. After incubating for the certain time point, for the DiI loaded liposomes, the liposome-plasma mixture was passed through Nanosep tubes with a 100 kD cut-off (Pall Life Sciences). The filtrate was collected, and for DiI, the fluorescence of the filtrate was measured with TECAN Infinite 200 Pro (Männedorf, Switzerland), while ICG the samples were measured at 780 nm.

### Cells

Mouse macrophages RAW 264.7 (Catalog no.: TIB-71, ATCC) and monocytic human THP-1 monocytes (Catalog no.: TIB-202, ATCC) were obtained from American Type Culture Collection (Rockville, MD). Cells were regularly tested for mycoplasma. 4T1-Luc mouse triple negative breast cancer cells were kindly provided by Dr. O van Tellingen (Netherlands Cancer Institute, Amsterdam, the Netherlands), originally received from Dr. Fred Miller at Karmanos Cancer Institute (Michigan), who originally developed these cells^42^. THP-1 were cultured in suspension in RPMI-1640 medium supplemented with 10% FBS (Lonza, Basel, Switzerland), 100 U/ml penicillin, 0.1 mg/ml streptomycin (Sigma Aldrich, St Louis, MO) and 2 mM l-glutamine (GE Healthcare, Little Chalfont, UK) in a CO_2_ incubator at 37 °C. Both RAW264.7 and 4T1 were cultured in RPMI-1640 medium, supplemented with 10% FBS, 2 mM l-glutamine, 100 U/ml penicillin and 0.1 mg/ml streptomycin.

### Macrophage differentiation

RAW264.7, M1 macrophages were differentiated using murine recombinant interferon-γ (IFNγ) and lipopolysaccharide (LPS), 10 ng/ml. M2 macrophages were differentiated using murine recombinant interleukin-4 (IL-4) and interleukin-13 (IL-13), 10 ng/ml. In case of THP-1 cells, THP-1 cells were treated for 24 h with 100 ng/ml of phorbol 12-myristate 13-acetate (PMA, Cayman chemicals) to allow their attachment. The medium was replaced by medium containing human recombinant INF-ϒ, 20 ng/ml and LPS, 100 ng/ml for M1 differentiation or human recombinant IL-4 and IL-13, 20 ng/ml for M2 differentiation. All cytokines were obtained from Peprotech, London, UK.

### Microscopic analysis of nanoliposome uptake

0.5 × 10^6^ THP-1 cells per well were seeded in a 12-well plate and differentiated as described above. Fluorescence of different nanoliposomes was measured in PBS, using the Victor3 multilabel fluorescence plate reader (Perkin Elmer, Waltham, MA). Nanoliposomes were diluted to equal fluorescence at a maximum concentration of 250 µM lipid in culture medium without serum and incubated for 2 h. After incubation, cells were vigorously washed and fixed for 20 min using 4% formalin. Cells were washed 2 times in PBS and mounted in mounting medium with DAPI. Uptake of nanoliposomes was visualized using the inverted fluorescent microscope EVOS (Applied Biosystems). Images were taken at 40× magnification using the fixed settings for the microscope.

### Quantitative nanoliposome uptake

Nanoliposomes were diluted to equal fluorescence at a maximum concentration of 250 µM lipid in culture medium without serum and added to differentiated macrophages in a 12-well plate and incubated at 37 °C for 2 h. After incubation, cells were vigorously washed, immediately placed on ice and then detached using Accutase cell detachment solution (Sigma Aldrich) and gentle scrapping. Cells were then collected and particle uptake was assessed by measuring mean fluorescence intensities in the FL-2 channel for at least 10,000 gated cells, using flow cytometry (BD FACS Calibur, Becton Dickinson, Franklin Lakes, NJ). Gates were set in the FSC vs SSC plot, using untreated control cell populations. For all experiments, identical settings were used. Data were analyzed using Flowing Software 2.5.0 and 2.5.1.

### Gene silencing studies

0.25 ×10^6^ THP-1 cells were plated in a 24-well plate and activated with PMA as described above. siRNA (CD36, Colec12, SR-B1, Thermo Fisher Scientific) complexes using HiPerfect Transfection reagent (Qiagen, Venlo, The Netherlands) were prepared as per the manufacturer’s instructions. siRNA was added to the cells at a concentration of 150 nM for 4 h, after which complexes were removed and cells were differentiated as described above. Cells were lysed 6 and 24 h after cytokine differentiation for PCR analysis. For quantitative nanoliposome uptake experiments, cells were used 44 h after cytokine differentiation.

### Cell viability

5 × 10^4^ THP-1 cells per well were seeded in a 96-well plate and activated using PMA as described above. Cells were then incubated in the presence or absence of serum for 24 h with different liposomal preparations (50 µM). After 24 h, medium was removed, cells were washed once with PBS, and a 10% 110 µg/ml Resazurin sodium salt solution (Sigma Aldrich) was added. Cells were allowed to incubate for an additional 75 minutes, after which the Resazurin solution (Alamar blue dye) was transferred to a black 96-well plate and fluorescence was measured using the Victor3 plate reader.

### Quantitative real-time PCR

Total RNA from cells was isolated using GenElute Mammalian Total RNA Miniprep Kit (Sigma Aldrich). RNA was isolated from mouse tumors and livers using the SV Total RNA isolation System (Promega, Madison, WI). cDNA was prepared by using iScript cDNA synthesis kit (Bio-Rad, Hercules, CA). Primer sequences are listed in Supplementary Table 4 and Supplementary Table 5. Reactions were measured using the CFX384 Real-Time PCR detection system (Bio-Rad). The threshold cycles (Ct) were calculated and relative gene expression was analyzed after normalization with the 18S or GAPDH as housekeeping genes.

### In vivo mouse tumor models

The experimental protocol was approved by the Central Commission for Animal Experiments of the Netherlands (ethical application numbers: 2013.III.03.024, AVD1100020174305). 6 weeks old female BALB/cAnNRj (18-20 g) were purchased from Charles River. Animals were housed in individually ventilated cages and fed ad libitum. The housing temperature was controlled at 20-24 °C and humidity of 40-70 % with a 12 h light and 12 h dark cycle was maintained. 1 × 10^5^ 4T1-luc cells (triple negative breast tumor cells) were injected into the mammary fat pad to develop orthotopic tumors. 5 × 10^5^ CT26 tumor cells (provided by Dr. Hawinkels, Leiden University Medical Centre, The Netherlands) were injected into the flank of the mice. Tumors were allowed to develop and the tumor size was determined using Vernier caliper and tumor volume was calculated using length × width × width × 0.52. Before sacrificing, 4T1 tumor bearing mice were injected with 3 mg of D-luciferin (Perkin Elmer, Waltham, MA) and were imaged after 10 min.

### In vivo distribution studies

Tissue and cellular localization: Female Balb/c mice were injected with 1 × 10^5^ 4T1 tumor cells into the mammary fat pad. Tumors were allowed to develop until tumor size reached 500 mm^3^. Liposomal formulations (HSPC-L, 0:8:2; PAPC-L, 3:5:2) containing fluorescent label DiI (equal fluorescence) were injected i.v. in mice. Mice were sacrificed after 1 h and organs and tumors were isolated. Equal amounts of frozen tissue from tumors, livers and spleens were lysed using RIPA buffer (Pierce). Tissues were homogenized thoroughly, centrifuged and fluorescence intensity in lysates was determined using the Victor 3 fluorescent plate reader. Fluorescence was corrected for protein content, determined using BCA kit (Thermo Fisher). Tissue sections from tumor, liver and spleen were cut and stained for macrophage marker F4/80 (Bio-rad) or CD206 (Santa Cruz) overnight. Secondary antibodies Rabbit anti Rat and Rabbit anti Goat (Dako) were used. Alexa 488 Donkey anti Rabbit (Thermo Fisher) antibodies were used for visualization. All antibodies are listed in Supplementary Table 6. Sections were scanned using the slide scanner (Nanozoomer, Hamamatsu).

### Near infrared (NIR) imaging

To study the total biodistribution, two different sets of experiments were performed. In 4T1 tumor-bearing mice (350 mm^3^ size), liposomal formulations containing ICG (equal fluorescence; equivalent to 1 nmol ICG dye/mouse) were injected intravenously in tumor-bearing mice (*n* = 3 per group) and mice were sacrificed after 2 h and 6 h. Organs and tumors were isolated for NIR imaging using Pearl imager (LICOR Inc.).

Flow cytometry analysis: Tumor-bearing mice (*n* = 3 per group) were injected with different liposomal formulations containing DiI dye and sacrificed after 2 h. Tumors were isolated and subjected to flow cytometry for further analysis. Then, they were cut into small pieces and placed in digestion buffer (RPMI containing 0.5 mg/ml of collagenase II and 0.5 mg/ml of DNAase) and subsequently incubated at 37 °C for 1 h (minced tissue was pipetted up and down around 20 times). Digested tissue was then centrifuged and washed with RPMI media. The tissue was again resuspended in digestion buffer containing 0.5 mg/ml of Dispase and incubated at 37 °C for 30 min. The digested cells were filtered through a 70 µm filter to remove undigested tissue and/or matrix proteins. Cells were fixed in 4% paraformaldehyde for 15 min at room temperature (RT), washed with PBS and then blocked with 5% BSA for 1 h. Then, cells were stained with different antibodies against cell surface markers in 0.5% BSA for overnight at 4 °C and washed with PBS. Later, cells were stained with fluorescently labeled secondary antibodies in 0.5% BSA for 1 h at RT. After staining, cells were washed and resuspended in PBS to analyze with BD FACS Calibur flow cytometer and the data was analyzed using Flow Jo ver. 7.6. The gating strategy is depicted in Supplementary Fig. 7a, b.

### In vivo efficacy studies

1 × 10^5^ 4T1-Luc cells were injected into the mammary fat pad or 5 × 10^5^ CT26 colon carcinoma cells were injected into the flank of the female balb/c mice. Tumors were allowed to develop. The tumor size was measured with Vernier caliper and tumor volume was calculated using length × width × width × 0.5. When tumor attain the size of 50-100 mm^3^, ZA-, AS- or MTP-loaded nanoliposomes (doses mentioned in the figure legends) were injected intravenously in 4T1 tumor-bearing mice, while ZA-nanoliposomes were also injected in CT26 tumor-bearing mice. The experimental end point of 1000 mm^3^ was maintained for all in vivo efficacy studies. Before sacrifice, 4T1 tumor-bearing mice were injected with 2.5 mg of d-luciferin (Perkin Elmer, Waltham, MA) and were imaged after 15 minutes to detect bioluminescence signal using Pearl Trilogy imager (LICOR).

### All-atom molecular dynamics simulations

Molecular dynamics (MD) simulations were performed using the Large-scale Atomic/Molecular Massively Parallel Simulator (LAMMPS) package^43,44^, which was successfully used in recent studies for the simulation of lipid bilayer^45–48^. The initial configurations were generated using a Monte-Carlo algorithm available in the Scienomics MAPS package^49,50^. The lipids were placed on two parallel planes, with straightened tails perpendicular to the planes, and surrounded with water molecules. Periodic boundary conditions were applied in all three directions. Interactions between all the atoms composing the water and lipids molecules were modeled by the all-atom Polymer-Consistent Force-Field (PCFF)^51^, using Ewald summation with an accuracy of 10^–3^ kcal/mol for electrostatic interactions, while van der Waals interactions were smoothly truncated at a distance of 15.5 Å. The generated system was energy minimized using the steepest descent method, followed by the conjugate gradient method. The system was next equilibrated for 1 ns in the isothermal-isobaric (*NPT*) ensemble at 298 K and 1 bar, followed by a 1 ns equilibration in the isothermal-isochoric (*NVT*) ensemble at 298 K, using the Nosé–Hoover thermostat^52,53^ and Berendsen barostat^54^. The production runs in the *NPT* ensemble covered 8 ns, with a 1 fs time step.

### Molecular docking

The docking of PAPC and HSPC with SR-B1 (model built-up based on PDB: 4F7B using Swiss-Model^35^), COLEC12 (PDB: 2OX8) and CD36 (PDB: 5LGD). The PDB file of the SR-B1 model is provided in the Supplementary data 1. Molecular docking was performed using Autodock mgltools version 1.5.6 default protocol. This version of Autodock helps us to predict the binding energy and binding pose of the compounds in detail. The Binding Energy = (1) + (2) + (3) − (4); whereas 1, 2, 3, 4 are defined as (1) Final Intermolecular Energy (vdW + Hbond + desolv Energy + Electrostatic Energy); (2) Final Total Internal Energy; (3) Torsional Free Energy; (4) Unbound System’s Energy. AutoDock4.2 is parameterized to use a model of the proteins and the ligands that includes polar hydrogen atoms, but not hydrogen atoms bonded to carbon atoms. An extended PDB format, termed PDBQT, is used for coordinate files, which includes atomic partial charges and atom types. Auto Dock Tools includes a number of methods for analyzing the results of docking simulations, including tools for clustering results by conformational similarity, visualizing conformations, interactions between ligands and proteins, and the affinity potentials created by Auto Grid. It employs two steps grid generation and docking. Grid generation builds a compound wall directing the ligand to bind in a particular area and the dimensions include *X*-axis = 126, *Y*-axis = 126 and *Z*-axis = 126 with the spacing of 0.375 and docking uses Lamarckian genetic algorithms which creates 2500000 evolutions and generates 27,000 docking poses and finally shows us the top 10 best-outfitted ligands with the protein. Interaction analyses were done using Bovia Discovery Studio Visualizer and all the interaction diagrams were obtained using Bovia Discovery Studio Visualizer software.

### Statistical analysis

Data are represented as the mean + standard error of the mean (SEM). The graphs and statistical analyses were performed using GraphPad Prism version 9.3.1. (GraphPad Prism Software, Inc., La Jolla, CA, USA). The type of statistical analysis has been mentioned in figure captions. The minimum significance difference was considered at *p* < 0.05.

### Reporting summary

Further information on research design is available in the Nature Research Reporting Summary linked to this article.

## Supplementary information

Supplementary Information

Description of Additional Supplementary Files

Supplementary Data 1

Supplementary Movie 1

Supplementary Movie 2

Supplementary Movie 3

Supplementary Movie 4

Reporting Summary

## Data Availability

The authors declare that all data supporting the findings of this study are available within the paper and its Supplementary Information files, including Supplementary Figs. 1–13. The supplementary videos 1–4 are available as separate files. The PDB file for the modeled SR-B1 and the PDB files for the docking of PAPC and HSPC with SR-B1 are provided in Supplementary Data 1 file. Supplementary Information accompanying this paper is appended. Source data are provided with this paper.

## Source data

Source Data
