# Supplementary material for: Cancer immune therapy using engineered ‛tail-flipping’ nanoliposomes targeting alternatively activated macrophages
Source: Nat Commun. 2022 Aug 4;13:4548. doi: 10.1038/s41467-022-32091-9 (PMC9352736; doi:10.1038/s41467-022-32091-9)
Supplement: Supplementary file 1 — Supplementary Information [file 41467_2022_32091_MOESM1_ESM.pdf]

Supplementary information

Cancer immune therapy using engineered 'tail-flipping' nanoliposomes targeting alternatively activated macrophages

Praneeth R. Kuninty[1,*], Karin Binnemars-Postma[1,*], Ahmed Jarray[2,3], Kunal P. Pednekar[1], Marcel A. Heinrich[1], Helen L. Pijffers[1], Hetty ten Hoopen[1], Gert Storm[1,4,5], Peter van Hoogevest[6], Wouter K. den Otter[2], Jai Prakash[1,#]

[1] Engineered Therapeutics, Department of Advanced Organ bioengineering and Therapeutics, TechMed Centre, University of Twente, Drienerlolaan 5, 7500AE, Enschede, The Netherlands

[2] Multi-scale Mechanics, Thermal and Fluid Engineering, Faculty of Engineering Technology, Drienerlolaan 5, 7500AE, University of Twente, Enschede, The Netherlands

[3] Physics and Physical Chemistry of Foods, Wageningen University, PO Box 17, 6700 AA Wageningen, The Netherlands

[4] Department of Pharmaceutics, Utrecht University, Universiteitsweg 99, 3584CG, Utrecht, The Netherlands

[5] Department of Surgery, Yong Loo Lin School of Medicine, C/O NUHS Tower Block, Level 8. IE Kent Ridge Road, National University of Singapore, Singapore

[6] Phospholipid Research Centre, Im Neuenheimer Feld 515, 69120 Heidelberg, Germany

* Equally Contributing Authors

[#]Corresponding author: j.prakash@utwente.nl

Supplementary information

Supplementary Figures

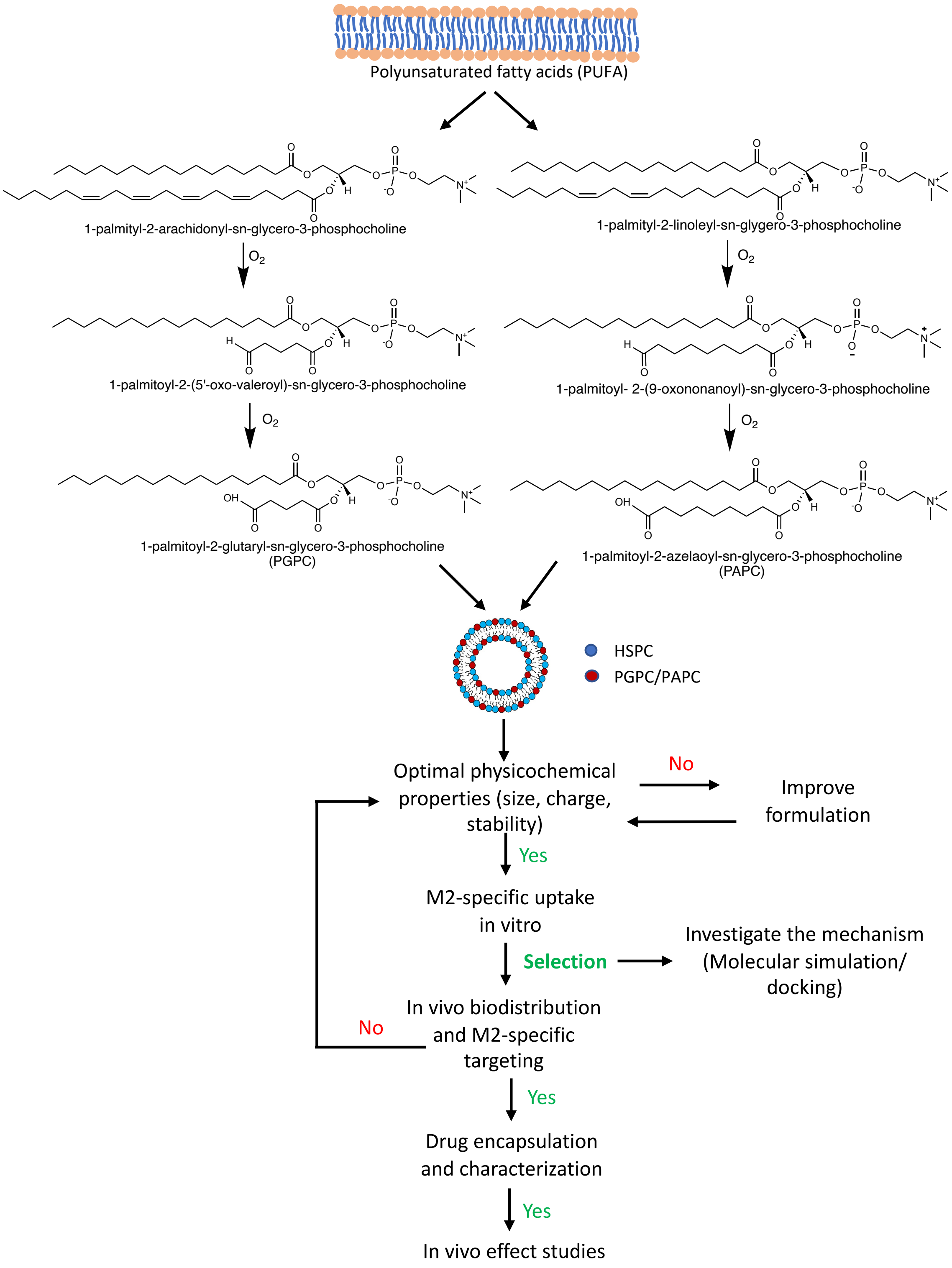

Supplementary Figure 1. Schematic diagram showing the synthesis of PAPC and PGPC lipids naturally and their selection procedure. PAPC and PGPC are generated from polyunsaturated fatty

acids (PUFA) as a result of cascade of oxidation process. They are saturated fatty acids with a stable carboxylated sn-2 tail. These lipids were incorporated into the nanoliposomes to target M2 macrophages. Using the following steps of characterization, in vitro uptake studies, mechanism investigation and biodistribution studies, the PAPC nanoliposomes were selected for drug targeting and in vivo efficacy studies.

Supplementary information

a

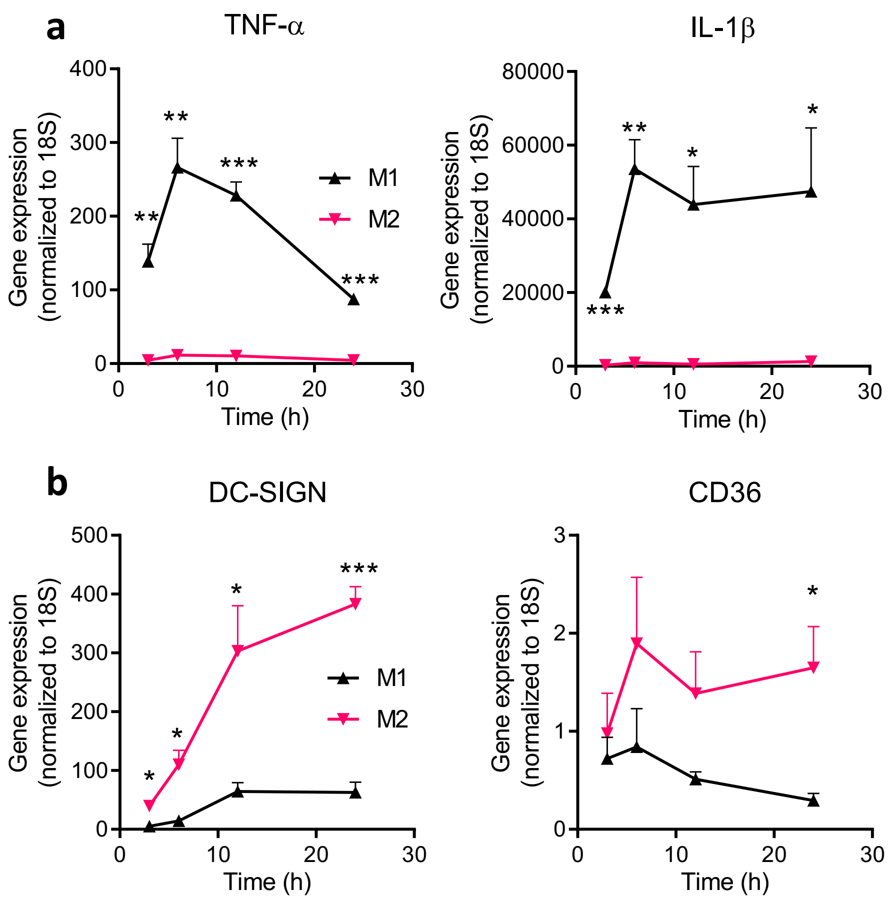

Supplementary Figure 2. Differentiation of M1 and M2 macrophages. THP-1 cells were treated with IFNγ + LPS to differentiate into M1 macrophages and with IL4/IL-13 to differentiate into M2 macrophages. Samples were collected at different timepoints and analyzed with qPCR for M1 markers (TNFα and IL-1β; **a)** and M2 markers (DC-SIGN, CD36; **b**). **(a, b)** Data represents mean + SEM for n=3 biologically independent experiments for all genes at 3, 6, and 12 hours while at 24 hours n=3 (TNF-α), n=4 (CD36, DC-SIGN), n=5 (IL-1β) (left to right, TNFα: **p=0.0049, **p=0.0030, ***p=0.00027, ***p=0.00016; IL-1β: ***p=0.000217, **p=0.0026, *p=0.013, *p=0.028; DC-SIGN: *p=0.012, *p=0.017, *p=0.038, ***p=0.000085; CD36: *p=0.018). Statistical analysis was performed with Multiple t-tests with correction for multiple comparisons using the Holm-Sidak's method. Source data are provided as a Source Data file.

Supplementary information

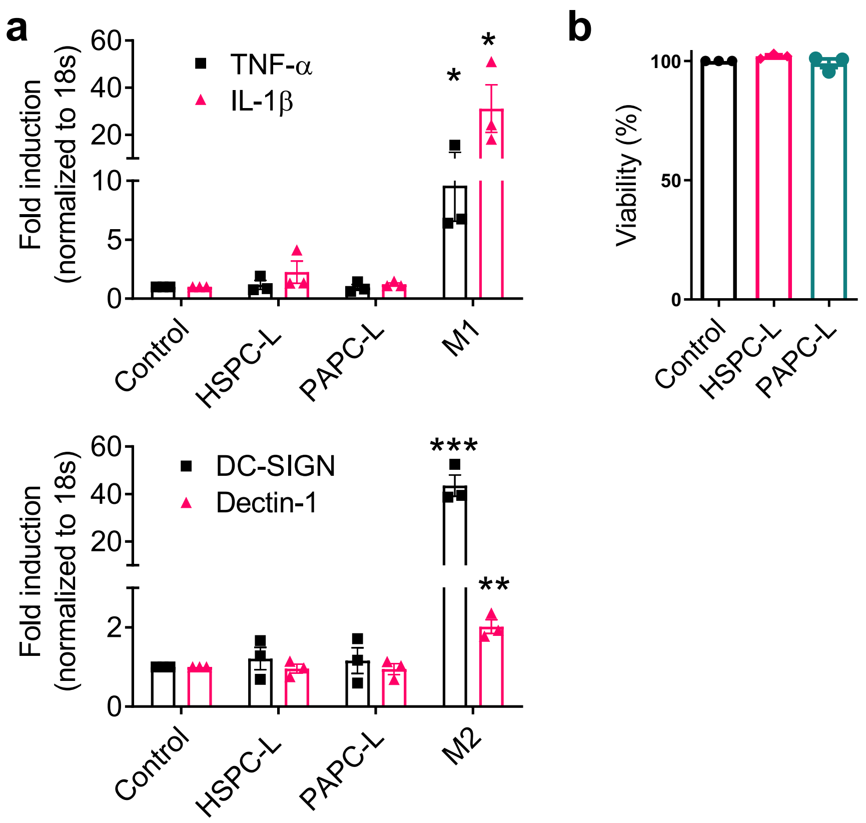

Supplementary Figure 3. Effect of HSPC-L and PAPC-L on the activation and viability of macrophages. **(a)** Gene expression data showing M1-associated genes (TNF-α, IL-1β) and M2-associated genes (DC-SIGN, Dectin-1) after the treatment of PMA-activated THP-1 with HSPC-L or PAPC-L (50 µM lipids) for 24 h. The positive controls for M1 and M2 were generated by the activation with LPS + IFNγ and IL4 + IL-13, respectively (TNF-α: *p=0.046; IL-1β: *p=0.040; DC-SIGN: ***p=0.00069; Dectin-1: **p=0.004 comparing control versus M2). Data represent the mean ± SEM from n=3 biologically independent experiments. Statistical analysis was performed with unpaired two-tailed Student's t-test. **(b)** % cell viability of PMA-differentiated macrophages, treated with HSPC-L or PAPC-L (50 µM lipids) for 24 h. Source data are provided as a Source Data file.

Supplementary information

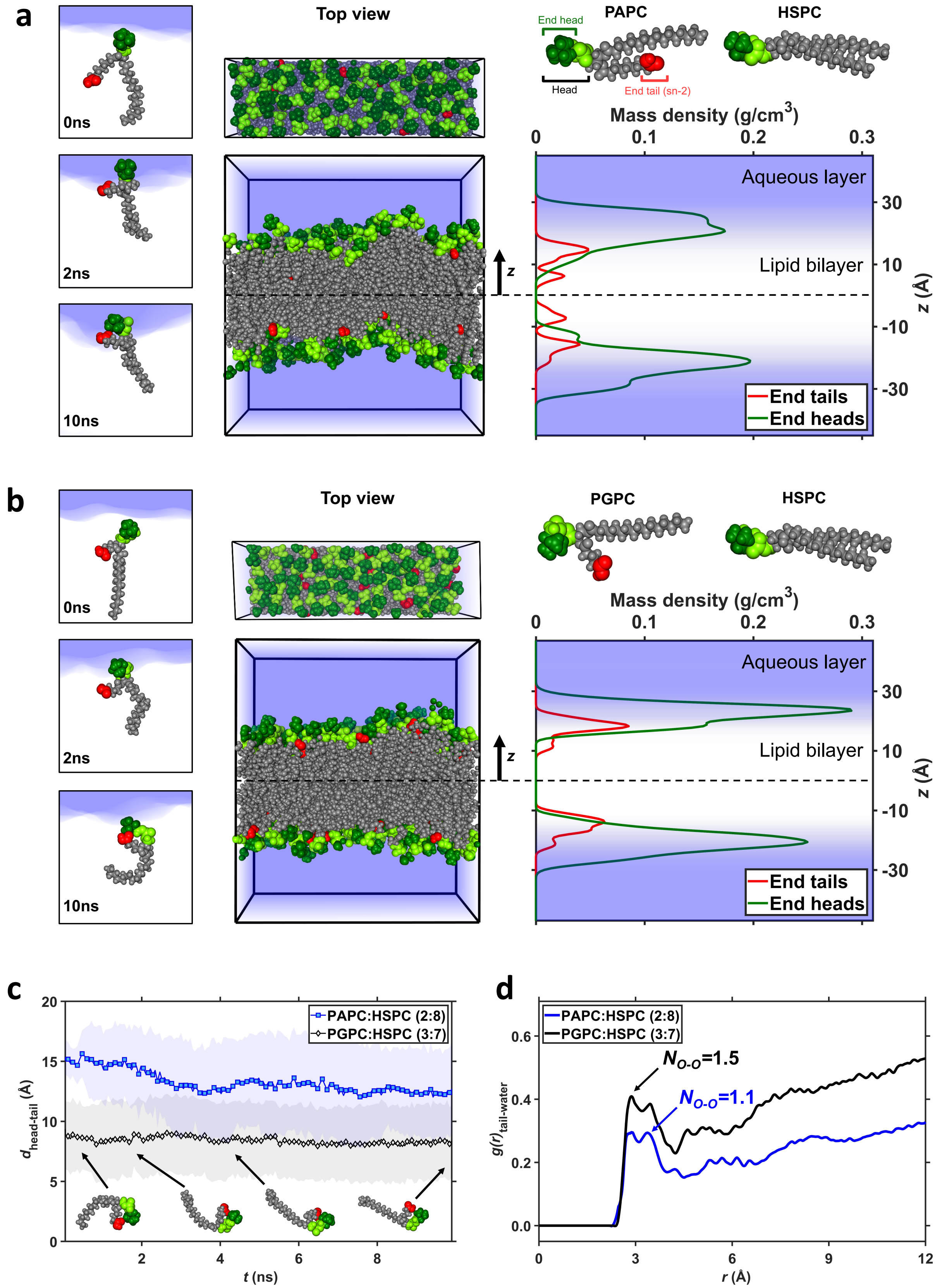

Supplementary Figure 4. Computation molecular simulation comparing “tail-flipping” phenomenon of PAPC-L and PGPC-L. Top and side views of **(a)** PAPC:HSPC (2:8) and (B) PGPC:HSPC (3:7) at the end of 10 ns molecular dynamics (MD) simulations, along with the mass density profiles of the tops of all lipid heads and sn-2 tail ends. Atoms in head groups are highlighted in dark green and the oxygen atoms in the sn-2 tails are colored in red. Their densities distributions along the bilayer normal are colored likewise. The blue zones depict aqueous regions. On the left side, snapshots of **(a)** PAPC and **(b)** PGPC lipid at the three times indicated, illustrating the locations of the sn-2 tail ends of the two lipids relative to heads. **(c)** The intra-molecular distance between the centers of mass of the head’s top and the sn-2 tail’s end of PAPC in PAPC/HSCP and of PGPC in PGPC/HSPC, plotted against simulation time. Average distances are represented by markers, standard deviations by the shaded areas. The simulations started with straightened tails; the tails with carboxylated ends subsequently folded to bring the oxygen atoms near the head group, while the all-carbon tails remained elongated. The snapshots show conformations of a PGPC lipid at the times and distances indicated by the arrow heads. **(d)** Radial distribution functions g(r) of water oxygen atoms relative to the two oxygen atoms at the end of the sn-2 tail of PAPC and PGPC, averaged over the last 1 ns of the simulation. Next to the curves are the average numbers $N_{O\text{-}O}$ and $N_{C\text{-}O}$ of contacts of per tail atom with oxygen atoms of water molecules in the first neighbor shell, i.e. contacts corresponding to the nearest-neighbor peak ending at 4.2 Å.

a

SRB1 sequence (aligned with LIMP-2 (PDB: 4F7B.1.A)

QMEANChain:A	PSLIKQQVLKNVRIDPSSLSFNMWKEIPIPFYLSVYFFDVMNPSEILKGEKPQVRERGPY
4f7b.1.A	-----QSIEKKIVLRNGTEAFDSWEKPPLPVYTQFYFFNVTNPEEILRGETPRVEEVGPY
QMEANChain:A	VYREFRHKSNITFNNN-DTVSFLEYRTFQFQPSKSHGS-ESDYIVMPNILVLGAAVMMEN
4f7b.1.A	TYRELRNKANIQFGDNGTTISAVSNKAYVFERDQSVGDPKIDLIRTLNIPVLTVIEWSQ-
QMEANChain:A	KPMTLKLIMTLAFTTLGERAFMNRTVGEIMWGYKDPLVNLINKYFPGMFPFKDKFGLFAE
4f7b.1.A	-VHFLREIIEAMLKAYQQKLFVTHTVDELLWGYKDEILSLIHVFRPDI----------SPYFGLFYE
QMEANChain:A	LNNSDSGLFTVFTGVQNISRIHLVDKWNGLSKVDFWHSDQCNMINGTSGQMWPPFMTPES
4f7b.1.A	KNGTNDGDYVFLTGEDSYLNFTKIVEWNGKTSLDWWITDKCNMINGTDGDSFHPLITKDE
QMEANChain:A	SLEFYSPEACRSMKLMYKESGVFEGIPTYRFVAPKTLFANGSIYPPNEGFCP----CLES
4f7b.1.A	VLYVFPSDFCRSVYITFSDYESVQGLPAFRYKVPAEILANT---SDNAGFCIPEGNCLGS
QMEANChain:A	GIQNVSTCRFSAPLFLSHPHFLNADPVLAEAVTGLHPNQEAHSLFLDIHPVTGIPMNCSV
4f7b.1.A	GVLNVSICKNGAPIIMSFPHFYQADERFVSAIEGMHPNQEDHETFVDINPLTGIILKAAK
QMEANChain:A	KLQLSLYMKSVAGIGQTGKIEPVVLPLLWFAESGAMEGETLHTFYTQLVLMPKVMHY
4f7b.1.A	RFQINIYVKKLDDFVETGDIRTMVFPVMYLNESVHIDKETASRLKSMI--------------------

b

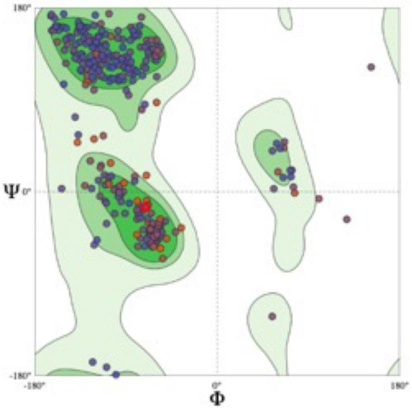

MolProbity Score	1.82	
Clash Score	4.78	(A293 GLU-_7 NAG), (A280 SER-_7 NAG), (A279 GLY-_7 NAG)
Ramachandran Favoured	94.15%	

c

Docking results showing binding energy (kcal/mol) from different runs

S. No.	Protein	Ligand	1st Run	2nd Run	3rd Run	4th Run	5th Run
1.	COLEC12 (PDB:2OX8)	HSPC	+4.39	+5.73	+6.61	+7.08	+7.37
2.		PAPC	-0.29	+1.09	+1.55	+1.94	+2.09
3.	CD36 (PDB:5LGD)	HSPC	+4.58	+4.90	+4.94	+5.15	+5.97
4.		PAPC	+0.04	+0.90	+1.84	+2.30	+2.97
5.	SR-B1 (built-up model)	HSPC	+3.70	+4.34	+4.46	+5.21	+5.29
6.		**PAPC**	**-2.97**	+1.25	+1.65	+1.66	+1.88

Supplementary Figure 5. Molecular docking of PAPC and HSPC with different receptors. (a) Sequences of SR-B1 (Uniport: Q8WTV0|33-443) and LIMP-2 (PDB: 4F7B.1.A). **(b)** Ramachandran plot showing the validation of the modeled SR-B1 generated by SWISS-MODEL based on the known LIMP-2 structure (PDB: 4F7B.1.A). **(c)** Docking results showing binding energy (kcal/mol) after different runs for the interaction of HSPC or PAPC with COLEC12 (PDB: 2OX8), CD36 (PDB: 5LGD) or SR-B1 (built-up model) using Autodock software.

Supplementary information

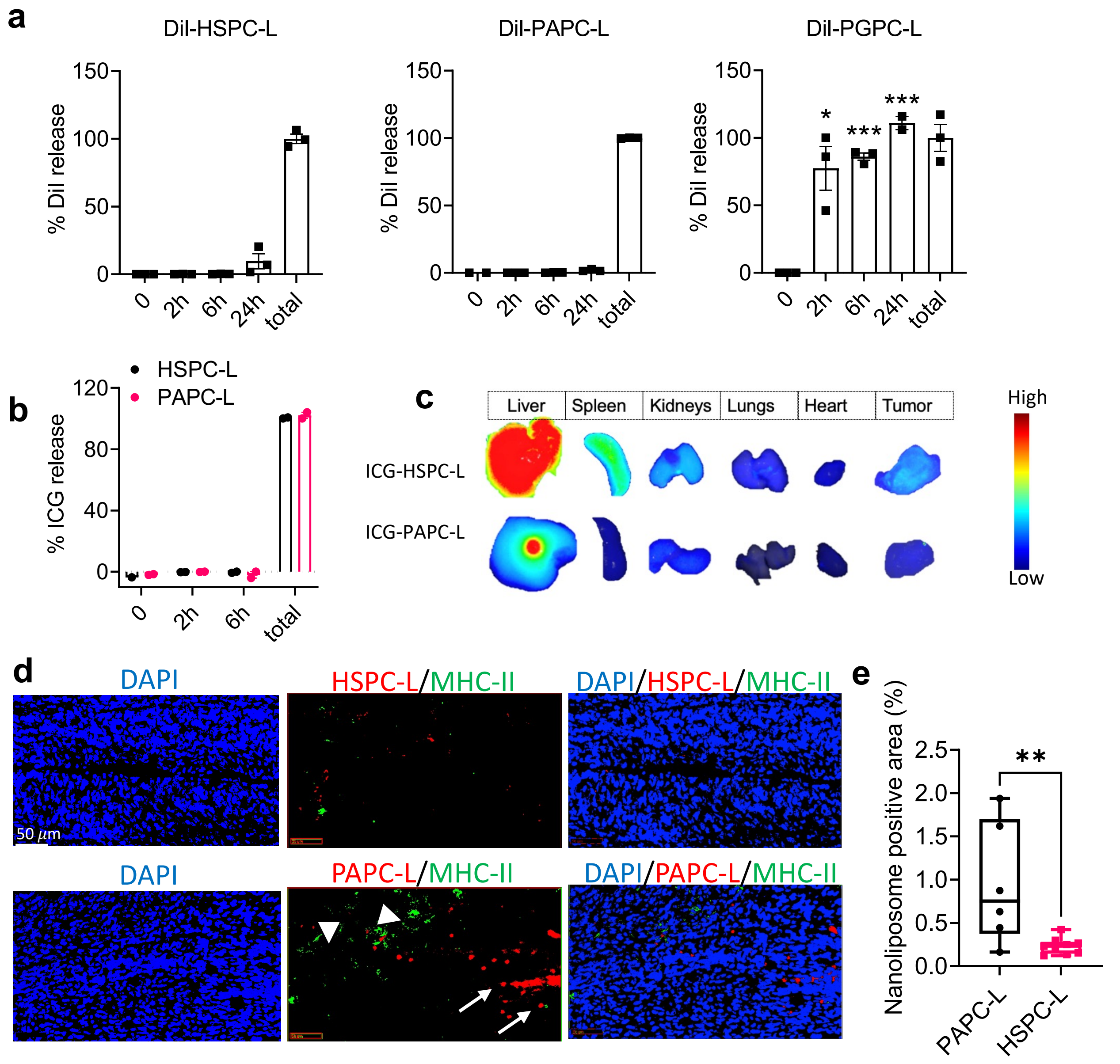

Supplementary Figure 6. Stability of dye containing nanoliposomes and distribution in vivo. (a) %DiI release after incubation of either DiI-HSPC-L, DiI-PAPC-L or DiI-PGPC-L in 50% plasma until 24 h. Total refers to the total amount of the encapsulated dye. Data represents mean ± SEM for three independent experiments. Statistical analysis was performed to compare DiI-PAPC-L versus DiI-PGPC-L using multiple unpaired t-test with correction for multiple comparisons using the Holm-Sidak's method. N=3, except for DiI-PGPC-L at 24h n=2 (left to right: *p=0.025, ***p=0.00003, ***p=0.00034)**. (b)** %ICG release after incubation of either DiI-HSPC-L or DiI-PAPC-L in 50% plasma until 6 h (maximum timepoint for the imaging studies). Data represents mean ± SEM for two

independent experiments. **(c)** Representative near-infrared images of different organs and tumor showing biodistribution of ICG-labeled HSPC-L and PAPC-L at t=6h. **(d)** Fluorescent microscopic images showing immunofluorescence staining of MHC-II (green) to localize with DiI-HSPC-L/PAPC-L (red color, arrows) with CD206+ TAMs (green color, arrowheads) within tumor tissues. Data show that there was hardly any co-localization of PAPC-L and MHC-II. **(e)** Box graph showing quantitative analysis of red color in the fluorescent tissue images shows that PAPC-L had a higher accumulation compared to HSPC-L in 4T1 tumor model. n=4 mice per group (**p=0.0084). Data represent the box-and-whisker plot where every dot represents a data point and the line in the box corresponds to the median. The boxes go from the upper to the lower quartiles, and the whiskers go from the minimum to the maximum value. Statistical analysis was performed with unpaired two-tailed student's t-test method. Source data are provided as a Source Data file.

Supplementary information

a

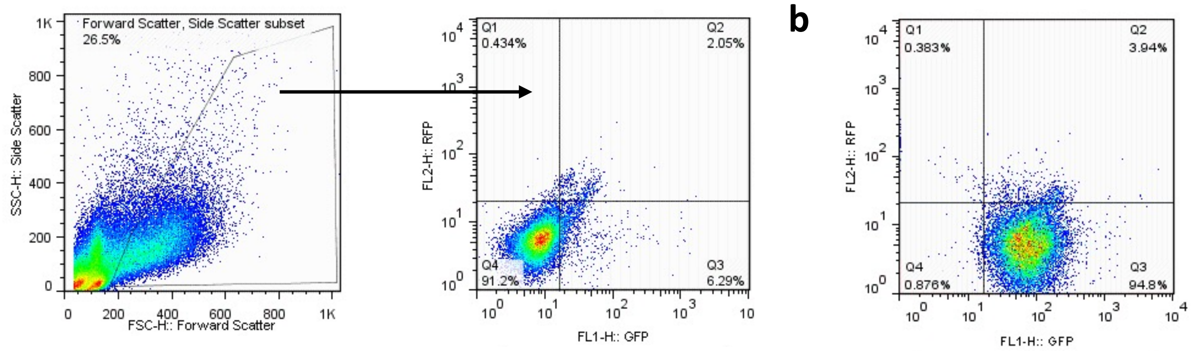

Supplementary Figure 7. Gating strategy for flow cytometry analysis. (a) Total lymphocytes were first gated on the FSC/SSC scatter, followed by gating on the secondary antibody control. **(b)** Gating strategy for cells showing 94.8% of them expressing CD68 (GFP channel) and 3.94% positive for DiI-labeled HSPC-L (RFP channel).

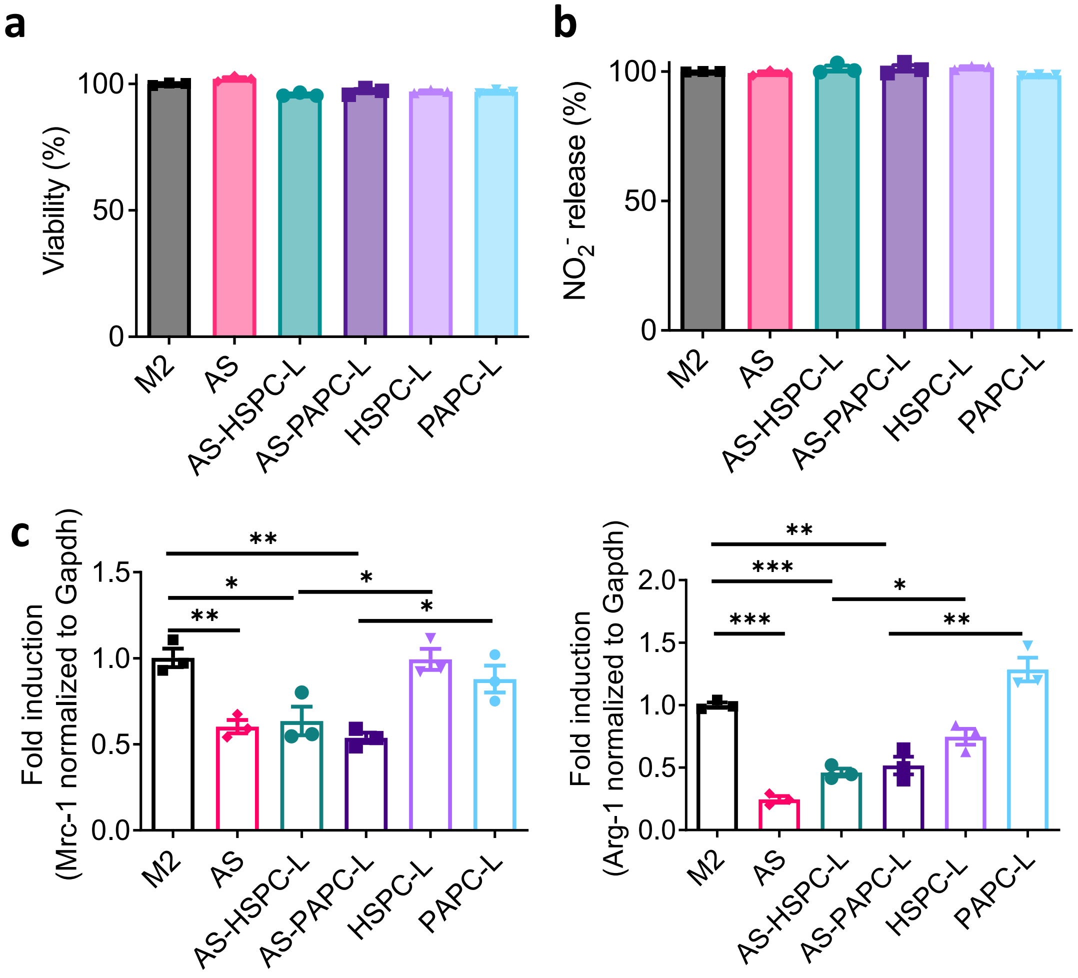

Supplementary Figure 8. Effect of AS-PAPC-L on M1 and M2 macrophages. (a-c) The effect of AS-HSPC-L and AS-PAPC-L were evaluated for their effect on cell viability using Alamar blue assay **(a)**, M1 differentiation, as shown with NO_2^- release assay **(b)** and on M2 differentiation, as shown with Mrc-1 and Arg-1 gene markers in M2 differentiated RAW macrophages **(c)** (left to right, Mrc-1: **p=0.0038, *p=0.020, **p=0.0016, *p=0.025, *p=0.015; Arg-1: ***p=0.000025, ***p=0.00014, **p=0.0028, *p=0.015, **p=0.0029). Data show that none of the treatments affected cell viability and NO_2^- release. Data are shown as mean ± SEM, n=3, Statistical analysis was performed with unpaired two-tailed student's t-test method. Source data are provided as a Source Data file.

Supplementary information

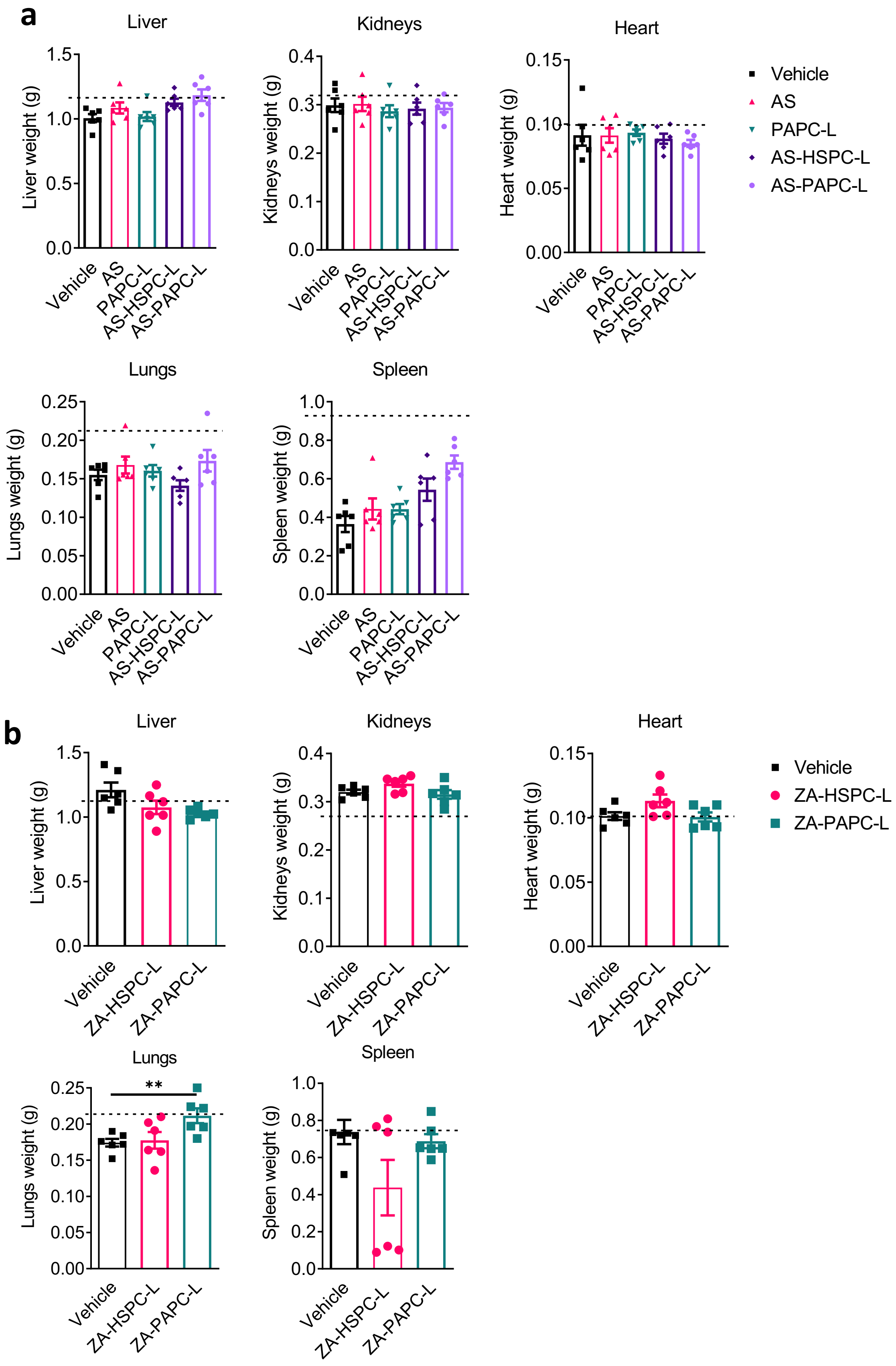

Supplementary Figure 9. Organ weights of mice treated with AS and ZA nanoliposomes. (a) Organ weights of mice treated with different treatments including empty PAPC-L, free AS, AS-HSPC-L and AS-PAPC-L in orthotopic 4T1 breast tumor model in mice. Free AS, AS-HSPC-L and AS-PAPC-L (dose equivalent to 8 mg/kg AS, 100 μl injection volume) were injected (free AS, i.p.; nanoliposomes, i.v.) twice a week when tumors became ±100 mm^3. Mean ± SEM, N=6 mice per group. **(b)** Organ weights of mice treated with different treatments including ZA-HSPC-L and ZA-PAPC-L in an orthotopic 4T1 breast tumor model in mice. Both ZA-HSPC-L and ZA-PAPC-L (dose equivalent to 0.5 mg/kg ZA, 100 μl injection volume) were injected intravenously twice a week when tumors became ±100 mm^3. Mean ± SEM, N=6 mice per group. The statistical analysis was performed using unpaired student's t-test method. (**p=0.0093). Source data are provided as a Source Data file.

Supplementary information

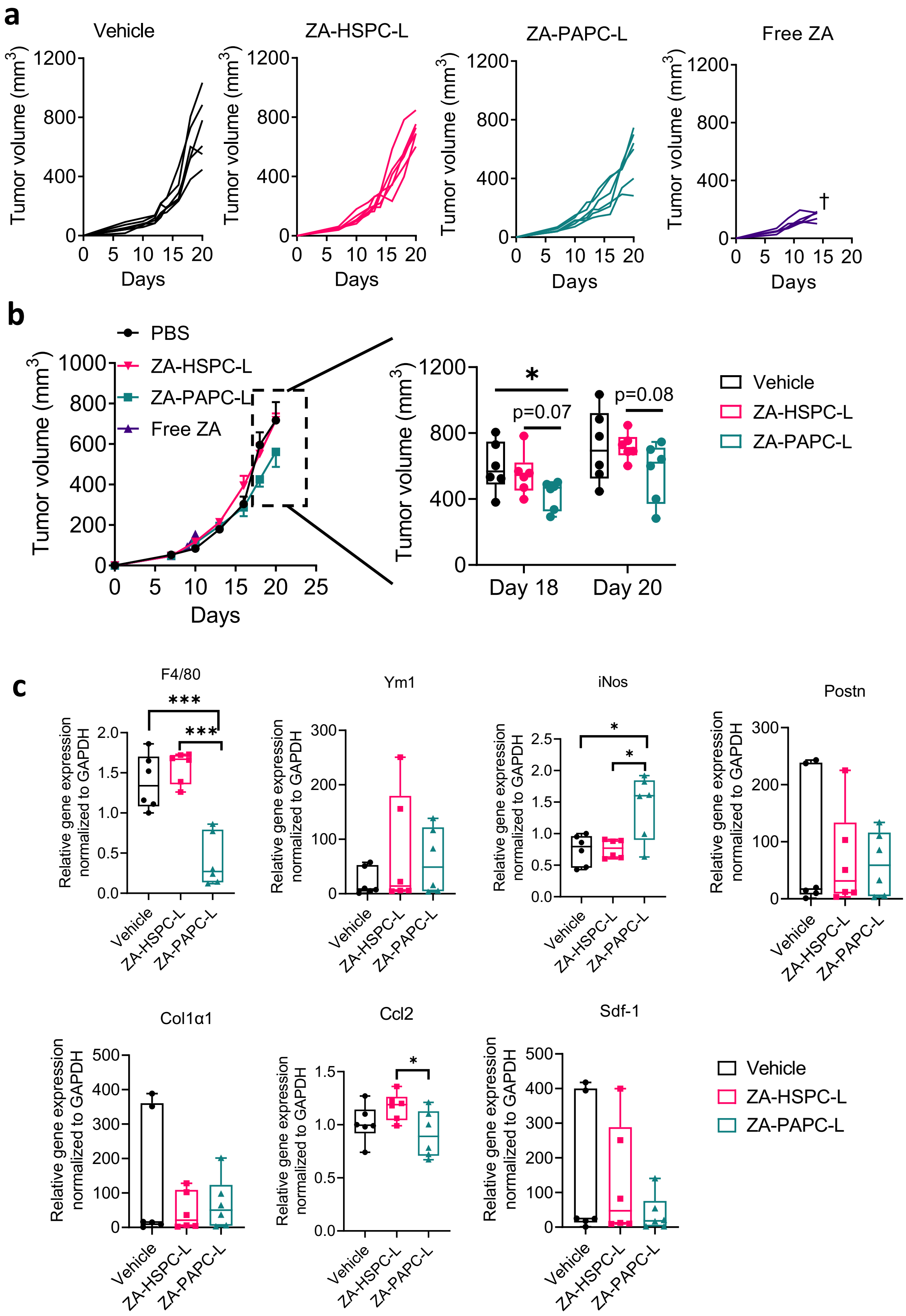

Supplementary information

Supplementary Figure 10. **The effect of ZA-HSPC-L and ZA-PAPC-L on the tumor growth in CT26 colon carcinoma model.** Individual **(a)** and mean **(b)** tumor growth curves showing the effect of ZA-HSPC-L and ZA-PAPC-L on the tumor growth in CT26 colon carcinoma tumor model in Balb/C mice. Both ZA-HSPC-L and ZA-PAPC-L (dose equivalent to 0.5 mg/kg ZA, 100 μl injection volume) were injected intravenously every third day when tumors became 50-100 mm^3. Tumor size was measured using Vernier Caliper (*p=0.040). Mean + SEM., N=6 mice per group. Right panel shows the box-and-whisker plot where every dot represents a data point and the line in the box corresponds to the median. The boxes go from the upper to the lower quartiles, and the whiskers go from the minimum to the maximum value. Statistical analysis was performed with unpaired two-tailed Student's t-test. † in Free ZA group indicates that the death of animals. **(c)** Box graphs showing quantitative real-time PCR results showing the effect of ZA-HSPC-L and ZA-PAPC-L on the markers for pre-metastatic niche in lungs (left to right, F4/80: ***p=0.00048, ***p=0.000020; iNos: *p=0.013, *p=0.011; Ccl2: *p=0.034). N=6 mice per group. Data represent the box-and-whisker plot where every dot represents a data point and the line in the box corresponds to the median. The boxes go from the upper to the lower quartiles, and the whiskers go from the minimum to the maximum value. Statistical analysis was performed with unpaired two-tailed Student's t-test. Source data are provided as a Source Data file.

Supplementary information

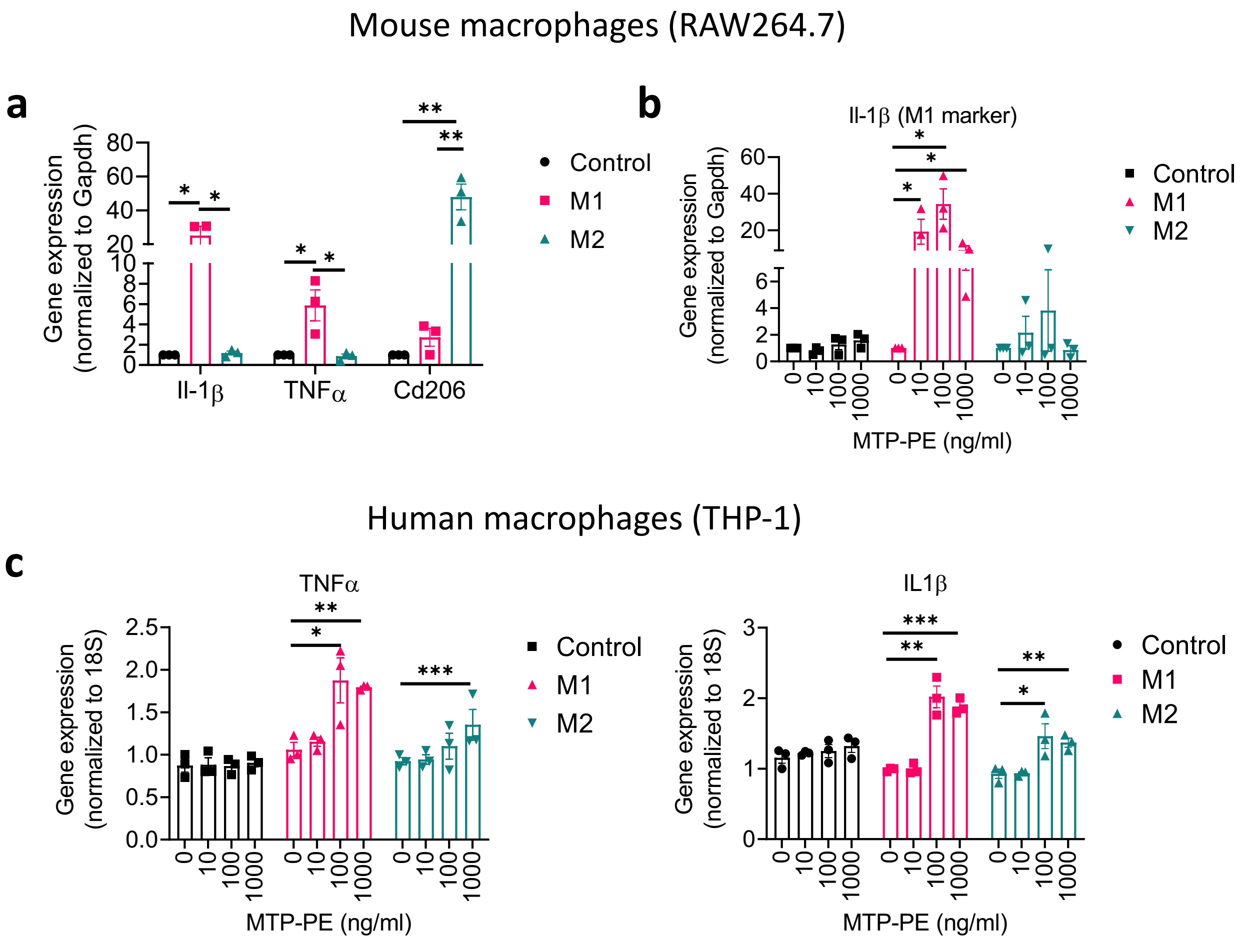

Supplementary Figure 11. Effect of MTP-PE on the activation of different macrophage phenotypes from human and mouse origin. (a) qPCR data showing the successful differentiation of RAW264.7 macrophages into M1 and M2 (left to right, Il-1β: *p=0.011, *p=0.011; Tnfα: *p=0.032, *p=0.031; Cd206: **p=0.0035, **p=0.0041). Data represents mean ± SEM for three independent experiments. Statistical analysis was performed with Multiple t-tests with correction for multiple comparisons using the Holm-Sidak's method. **(b)** The gene expression data showing the effect of MTP-PE with the increasing concentrations on control, M1 and M2 differentiated cells for IL-1β (left to right: *p=0.015, *p=0.026). Bars represent mean ± SEM, n=3. Unpaired two-tailed student's t test. **(c)** qPCR analysis showing the effect of MTP-PE on THP-1 after the treatment with PMA (control) and M1 and M2 differentiated cells for M1 markers IL-1β and TNFα (left to right, TNFα: *p=0.043, **p=0.0010, ***p=0.00078; IL1β: **p=0.0026, ***p=0.00017, *p=0.046, **p=0.0083). A) MTP-PE treatment inhibits M2 marker and activates tumoricidal properties by upregulating M1 markers. B) The cell growth was evaluated after 24 h treatment of MTP-PE and INFϒ C) MTP-PE, in

synergy with INFϒ activates tumoricidal properties by inducing more M1 markers. Bars represent mean + SEM, n=3. Unpaired two-tailed student's t test. Source data are provided as a Source Data file.

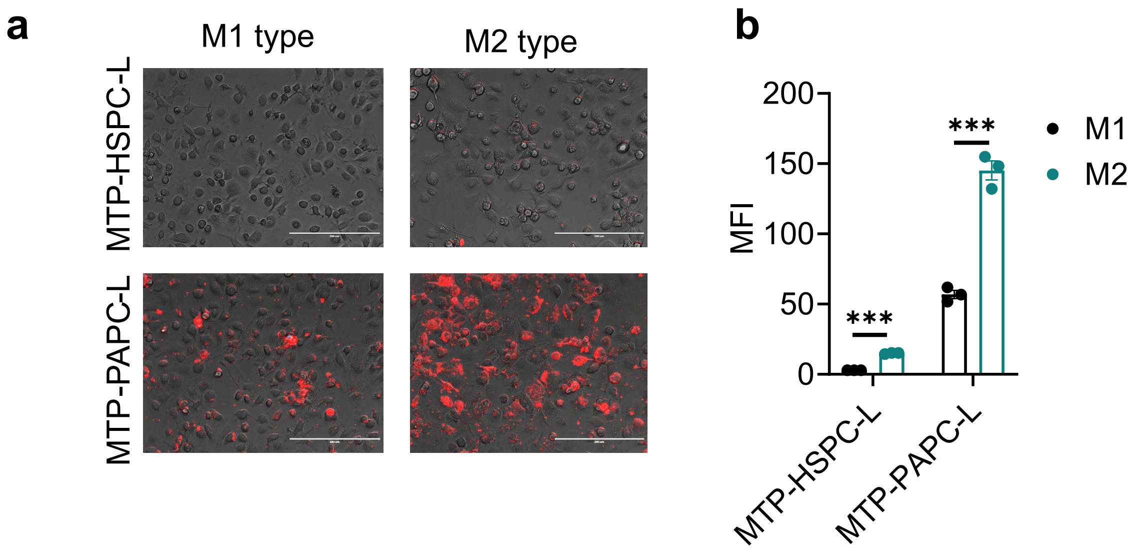

Supplementary Figure 12. Uptake of MTP-HSPC-L and MTP-PAPC-L in M1 and M2 macrophages. **(a)** Microscopic images and **(b)** quantitation showing the cellular uptake of MTP-HSPC-L and MTP-PAPC-L labeled with DiI dye (red color) in M1 and M2 macrophages (left to right: ***p=0.000001, ***p=0.00027). Scale bar: 200 μm. Data represents mean ± SEM for three independent experiments. Statistical analysis was performed with unpaired two-tailed Student's t-test. Source data are provided as a Source Data file.

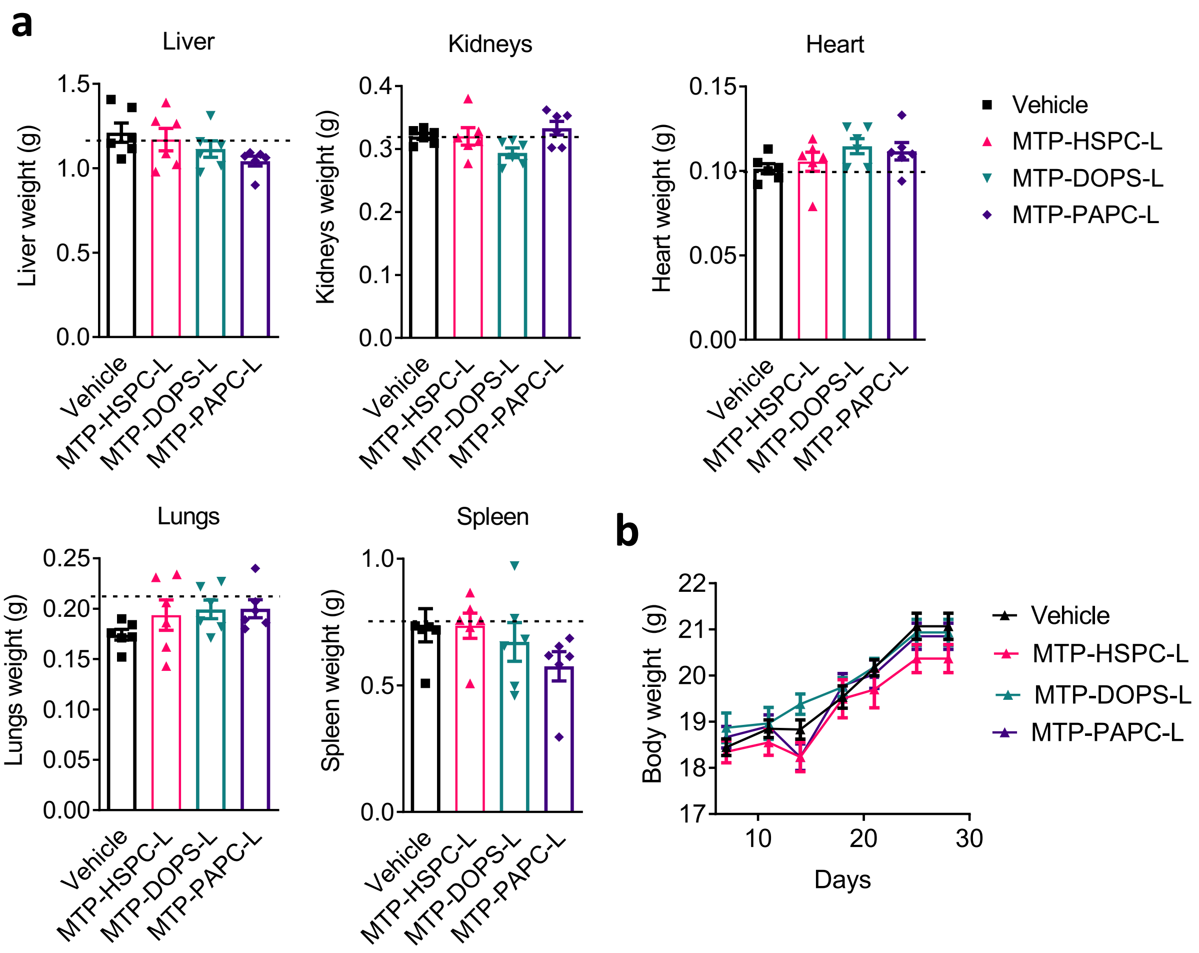

Supplementary Figure 13. Organ and body weights of mice treated with MTP nanoliposomes. Organ **(a)** and body **(b)** weights of mice treated with different treatments including vehicle, MTP-HSPC-L, MTP-DOPC-L or MTP-PAPC-L in orthotopic 4T1 breast tumor model. All formulations had a dose of MTP-PE equivalent to 1 mg/kg/mouse, 100 μl injection volume. Mean + SEM., N=6 mice per group. Source data are provided as a Source Data file.

Supplementary information

Supplementary Tables

Supplementary Table 1: **Size, polydispersity index (PDI) and zeta potential of different compositions of nanoliposomes.**

**Nanoliposomes**	**Size (nm ± SD)**	**PDI (± SD)**	**Zeta potential (mV ± SD)**
HSPC-L (8:2)	115.9 ± 6.2	0.12 ± 0.05	-22.6 ± 4.8
PAPC-L (2:6:2)	107.9 ± 0.7	0.19 ± 0.06	-25.1 ± 4.9
PAPC-L (3:5:2)	105.9 ± 15.7	0.22 ± 0.04	-27.8 ± 4.6
PGPC-L (2:6:2)	80.1 ± 0.9	0.20 ± 0.10	-17.0 ± 6.0
PGPC-L (3:5:2)	113.5 ± 16.5	0.2 ± 0.08	-21.1 ± 2.8

Molar ratios are shown between brackets. Results are shown from 2 to 5 different batches of nanoliposomes. Standard deviation (SD). HSPC nanoliposomes (HSPC-L, HSPC:Cholesterol=8:2), PAPC-L (PAPC:HSPC:Cholesterol= 2:6:2; 3:5:2) and PGPC-L (PGPC:HSPC:Cholesterol= 2:6:2; 3:5:2).

Supplementary information

Supplementary Table 2: Analysis of lipid content in nanoliposomes.

**Nanoliposomes**	**PAPC/PGPC (mM)**	**HSPC (mM)**	**Cholesterol (mM)**	**Calculated molar ratio**
HSPC-L (8:2)	-	1.6 (2)	0.4 (0.5)	8:2
PAPC-L (3:5:2)	0.6 (0.75)	1.0 (1.25)	0.4 (0.5)	3:5:2
PGPC-L (3:5:2)	0.4 (0.75)	0.9 (1.25)	0.4 (0.5)	2.4: 5.3: 2.4

Data showing lipid content analyzed using ultra-high performance liquid chromatography (uHPLC) with corona charged aerosol detector (CAD). Values are shown in mM, with theoretical amounts between brackets. The calculated molar ratio according to uHPLC analysis is shown in the last column. HSPC nanoliposomes (HSPC-L, HSPC:Cholesterol=8:2), PAPC-L (PAPC:HSPC:Cholesterol= 3:5:2) and PGPC-L (PGPC:HSPC:Cholesterol= 3:5:2)

Supplementary information

Supplementary Table 3: **Change in size of a typical batch of nanoliposomes after long storage at 4°C for 3 weeks.**

**Nanoliposomes**	**Size (nm ± SD) T=0**	**PDI (± SD) T=0**	**Size (nm ± SD) T=3 weeks**	**PDI (± SD) T=3 weeks**
HSPC-L (0:8:2)	116.1 ± 3.36	0.06 ± 0.01	118.0 ± 0.36	0.12 ± 0.01
PAPC-L (3:5:2)	83.34 ± 3.34	0.17 ± .003	87.23 ± 2.94	0.22 ± 0.01
PGPC-L (3:5:2)	96.79 ± 2.57	0.14 ±1.5	91.9 ± 0.44	0.16 ± 0.03

Polydispersity index (PDI), standard deviation (SD). HSPC nanoliposomes (HSPC-L, HSPC:Cholesterol=8:2), PAPC-L (PAPC:HSPC:Cholesterol=3:5:2) and PGPC-L (PGPC:HSPC:Cholesterol=3:5:2)

Supplementary information

Supplementary Table 4: Primer sequences for human genes

Gene	Forward (5'→ 3')	Reverse (3'→ 5')	Accession code
RPS18	TGAGGTGGAACGTGTGATCA	CCTCTATGGGCCCGAATCTT	NM_022551.2
DC-SIGN	GAACTGGCACGACTCCATCA	CTGGAAGACTGCTCCTCAGC	NM_001144897.1
DECTIN-1	ATGGCTCTGGGAGGATGGAT	GGGCACACTACACAGTTGGT	NM_197947.2
TNF-α	CTTCTGCCTGCTGCACTTTG	GTCACTCGGGGTTCGAGAAG	NM_000594.3
IL-1β	CAGAAGTACCTGAGCTCGCC	AGATTCGTAGCTGGATGCCG	NM_000576.2
IL-6	TGCAATAACCACCCCTGACC	ATTTGCCGAAGAGCCCTCAG	NM_000600.3
CD36	TGGCAACAAACCACACACTG	AAGTCCTACACTGCAGTCCTC	NM_000072.3
Colec12	AGGTCGAGGTTAGACACTGAAG	GATCCTCTGTCACCTCTTGGAC	NM_130386.2
Scarb1	AAGATTGAGCCTGTGGTCCTG	CCTCCTTATCCTTTGAGCCCT	NM_005505.4

Supplementary information

Supplementary Table 5: Primer sequences for mouse genes

Gene	Forward	Reverse	Accession code
F4/80	TGCATCTAGCAATGGACAGC	GCCTTCTGGATCCATTTGAA	NM_010130.4
Gapdh	ACAGTCCATGCCATCACTGC	GATCCACGACGGACACATTG	XM_001476707.3, XM_001479371.4, XM_003946114.1, NM_008084.2
Il-1β	GCCAAGACAGGTCGCTCAGGG	CCCCCACACGTTGACAGCTAGG	NM_008361.3
Il-6	TGATGCTGGTGACAACCACGGC	TAAGCCTCCGACTTGTGAAGTG GTA	NM_031168.1
Mrc -1	GGGACGTTTCGGTGGACTGTGG	TTGTGGGCTCTGGTGGGCGA	NM_008625.2
Postn	ATCCACGGAGAGCCAGTCAT	TGTTTCTCCACCTCCTGTGG	NM_001198766.1
Ym-1	ACTTTGATGGCCTCAACCTG	AATGATTCCTGCTCCTGTGG	NM_009892.2
Col 1a1	TGACTGGAAGAGCGGAGAGT	ATCCATCGGTCATGCTCTCT	NM_007742.3
CCR2	GCCATCATAAAGGAGCCATACC	ATGCCGTGGATGAACTGAGG	NM_009915.2
CCl2	GTGCTGACCCCAAGAAGGAA	GTGCTGAAGACCTTAGGGCA	NM_011333.3
SDF1a	CAGAGCCAACGTCAAGCA	AGGTACTCTTGGATCCAC	NM_001012477.2, NM_013655.4, NM_021704.3
iNOS	AATCTTGGAGCGAGTTGTGG	CAGGAAGTAGGTGAGGGCTTG	XM_006532446.1; NM_010927.3

Supplementary information

Supplementary Table 6: Information about antibodies used for immunostaining and flow cytometry

Antibody	Host	Mono/ Polyclonal	Source	Cat. no.	Clone no.	Dilution ratio
CD206	goat	Poly	Santa cruz	sc-34577	C-20	1:100
CD68	rabbit	poly	santa cruz	sc-9139	H-255	1:100
CD86	rabbit	mono	Novus biologicals	NB110-55488	EP1158Y	1:50
Ym1	Goat	poly	R & D systems	BAF2446		1:50
CD36	Rabbit	Mono	Abcam	ab133625	EPR6573	1:50
MHC-II	Rat	Mono	Santa cruz	sc-59318	ER-TR3	1:100
Alexa Fluor® 488 Donkey Anti-Goat IgG	Donkey		Life technologies	A-11055		1:100
Alexa Fluor® 488 Donkey anti-Rabbit IgG	Donkey		Life technologies	A-21206		1:100
