# Supplementary material for: Cancer immune therapy using engineered ‛tail-flipping’ nanoliposomes targeting alternatively activated macrophages
Source: Nat Commun. 2022 Aug 4;13:4548. doi: 10.1038/s41467-022-32091-9 (PMC9352736; doi:10.1038/s41467-022-32091-9)
Supplement: Supplementary file 2 — Description of Additional Supplementary Files [file 41467_2022_32091_MOESM2_ESM.pdf]

Description of Additional Supplementary Files

File Name: Supplementary Data 1
Description: PDB files

File Name: Supplementary Movie 1
Description: All-atom molecular dynamics simulations of PAPC: HSPC (3:7) bilayer system. (A) side view, and (B) individual PAPC lipid. Atoms in head groups are highlighted in dark green, the oxygen atoms at the end of the sn-2 tails are coloured in red, and the carbon atoms at the end of the sn-1 tails are coloured in black. Total simulation time is 10 ns.

File Name: Supplementary Movie 2
Description: All-atom molecular dynamics simulations of pure HSPC bilayer system. (A) side view, and (B) individual HSPC lipid. Atoms in head groups are highlighted in dark green, and the carbon atoms at the end of the sn-1 tails are coloured in black. Total simulation time is 10 ns.

File Name: Supplementary Movie 3
Description: All-atom molecular dynamics simulations of PAPC: HSPC (2:8) bilayer system. (A) side view, and (B) individual PAPC lipid. Atoms in head groups are highlighted in dark green, the oxygen atoms at the end of the sn-2 tails are coloured in red, and the carbon atoms at the end of the sn-1 tails are coloured in black. Total simulation time is 10 ns.

File Name: Supplementary Movie 4
Description: All-atom molecular dynamics simulations of PGPC: HSPC (3:7) bilayer system. (A) side view, and (B) individual PGPC lipid. Atoms in head groups are highlighted in dark green, the oxygen atoms at the end of the sn-2 tails are coloured in red, and the carbon atoms at the end of the sn-1 tails are coloured in black. Total simulation time is 10 ns.
