# Supplementary material for: Cancer immune therapy using engineered ‛tail-flipping’ nanoliposomes targeting alternatively activated macrophages
Source: Nat Commun. 2022 Aug 4;13:4548. doi: 10.1038/s41467-022-32091-9 (PMC9352736; doi:10.1038/s41467-022-32091-9)
Supplement: Supplementary file 8 — Reporting Summary [file 41467_2022_32091_MOESM8_ESM.pdf]

nature research

Corresponding author(s): Jai Prakash

Last updated by author(s): 07-06-2022

Reporting Summary

Nature Research wishes to improve the reproducibility of the work that we publish. This form provides structure for consistency and transparency in reporting. For further information on Nature Research policies, see our Editorial Policies and the Editorial Policy Checklist.

Statistics

For all statistical analyses, confirm that the following items are present in the figure legend, table legend, main text, or Methods section.

n/a	Confirmed	
☐	☒	The exact sample size (*n*) for each experimental group/condition, given as a discrete number and unit of measurement
☐	☒	A statement on whether measurements were taken from distinct samples or whether the same sample was measured repeatedly
☐	☒	The statistical test(s) used AND whether they are one- or two-sided<br>*Only common tests should be described solely by name; describe more complex techniques in the Methods section.*
☐	☒	A description of all covariates tested
☐	☒	A description of any assumptions or corrections, such as tests of normality and adjustment for multiple comparisons
☐	☒	A full description of the statistical parameters including central tendency (e.g. means) or other basic estimates (e.g. regression coefficient) AND variation (e.g. standard deviation) or associated estimates of uncertainty (e.g. confidence intervals)
☐	☒	For null hypothesis testing, the test statistic (e.g. *F*, *t*, *r*) with confidence intervals, effect sizes, degrees of freedom and *P* value noted<br>*Give P values as exact values whenever suitable.*
☒	☐	For Bayesian analysis, information on the choice of priors and Markov chain Monte Carlo settings
☒	☐	For hierarchical and complex designs, identification of the appropriate level for tests and full reporting of outcomes
☒	☐	Estimates of effect sizes (e.g. Cohen's *d*, Pearson's *r*), indicating how they were calculated

Our web collection on statistics for biologists contains articles on many of the points above.

Software and code

Policy information about availability of computer code

Data collection	Molecular docking was performed using Autodock software mgltools version 1.5.6.<br>The protein models were designed using Swiss-Model (https://swissmodel.expasy.org).<br>Molecular dynamics simulations were performed using the Large-scale Atomic/Molecular Massively Parallel Simulator package (LAMMPS) which is an open-source package for performing classical molecular dynamics simulations with a focus on materials modeling (https://lammps.sandia.gov/). MAPS is a commercial modeling platform for generating input data for LAMMPS and for moedling materials (https://www.scienomics.com/maps-platform/)"<br>Flow cytometry data was collected with BD CellQuest Pro ver. 5.2.1.
Data analysis	The graphs and statistical analyses were performed using GraphPad Prism version 9.3.1. (GraphPad Prism Software, Inc., La Jolla, CA, USA).<br>Protein structures were visualized using Bovia Discovery Studio Visualizer.<br>Flow cytometry analysis for in vivo analysis was performed using Flow Jo ver. 7.6 while Flowing software ver. 2.5.0 and 2.5.1 was used for in vitro data analysis.<br>The graphs and statistical analyses were performed using GraphPad Prism version 9.3.1. (GraphPad Prism Software, Inc., La Jolla, CA, USA).

For manuscripts utilizing custom algorithms or software that are central to the research but not yet described in published literature, software must be made available to editors and reviewers. We strongly encourage code deposition in a community repository (e.g. GitHub). See the Nature Research guidelines for submitting code & software for further information.

Data

Policy information about availability of data

All manuscripts must include a data availability statement. This statement should provide the following information, where applicable:

- Accession codes, unique identifiers, or web links for publicly available datasets
- A list of figures that have associated raw data
- A description of any restrictions on data availability

The docking of PAPC and HSPC with SR-B1 (model built-up based on PDB: 4F7B using Swiss-Model36), COLEC12 (PDB: 2OX8) and CD36 (PDB: 5LGD).
The following accession numbers were used for designing primers.
RPS18 - NM_022551.2
DC-SIGN - NM_001144897.1
DECTIN-1 - NM_197947.2
TNF-α - NM_000594.3
IL-1β - NM_000576.2
IL-6 - NM_000600.3
CD36 - NM_000072.3
Colec12 - NM_130386.2
Scarb1 - NM_005505.4
F4/80 - NM_010130.4
Gapdh - XM_001476707.3, XM_001479371.4, XM_003946114.1, NM_008084.2
Il-1β - NM_008361.3
Il-6 - NM_031168.1
Mrc -1 - NM_008625.2
Postn - NM_001198766.1
Ym-1 - NM_009892.2
Col 1a1 - NM_007742.3
CCR2 - NM_009915.2
CCl2 - NM_011333.3
SDF1a - NM_001012477.2, NM_013655.4, NM_021704.3
iNOS - XM_006532446.1; NM_010927.3, NM_005505.4

PDB codes for LIMP-2 (PDB: 4F7B), COLEC12 (PDB: 2OX8) and CD36 (PDB: 5LGD) are hyperlinked.
PDB files of the generated SR-B1 model and PDB files for docking of PAPC and HSPC with SR-B1 are added to Suppl. Data 1.

The source data for the figures 1b, 1d, 1f, 1g, 2a-d, 5c-f, 5h, 6c, 6e, 7c, 7d, 7f, 7g, 8b, 8d-e, 8g-h, 9, and supplementary figures S2, S3, S6, S8, S9, S10, S11, S12, S13 are provided with this paper as a source data file.
The molecular dynamic simulation graphs were generated by the used software and no raw data is therefore available.
Chemdraw files for all chemical structures are provided separately.

Field-specific reporting

Please select the one below that is the best fit for your research. If you are not sure, read the appropriate sections before making your selection.

☒ Life sciences ☐ Behavioural & social sciences ☐ Ecological, evolutionary & environmental sciences

For a reference copy of the document with all sections, see nature.com/documents/nr-reporting-summary-flat.pdf

Life sciences study design

All studies must disclose on these points even when the disclosure is negative.

Sample size	For animal studies, the sample size was determined using Power analysis based on the %CV and meaningful differences to reach the minimum significance level of p<0.05. For in vitro studies, the sample size was not pre-determined but all experiments were performed minimally n=3 independent experiments. The sample size was determined based on the percentage variation observed within an assay and we have found that n=3 in most cases was sufficient to find at least the minimal significance level of p<0.05 with two-sided unpaired student t-test.
Data exclusions	No data was excluded in this study.
Replication	All in vitro experiments were performed as at least n=3 independent experiments as described in the figure legends. For some assays such as cell viability assay, each experiment had 2-3 technical replicates which were averaged and considered as one experiment.<br>In vivo experiments as performed with the sample size are included in the experiments. Experimental findings were reliably reproduced between different experiments and some experiments were replicated by different researchers involved within the study.
Randomization	For in vivo studies, the tumor-bearing mice were randomized for different treatment groups in an unbiased manner. After tumors were palpable, they were divided into different cohorts based on the tumor size and then animals were distributed into different groups from each cohort to reach the similar tumor size in each group.
Blinding	Investigators were not blinded as the administrations, sample collection and processing were conducted by the same researchers.

Reporting for specific materials, systems and methods

We require information from authors about some types of materials, experimental systems and methods used in many studies. Here, indicate whether each material, system or method listed is relevant to your study. If you are not sure if a list item applies to your research, read the appropriate section before selecting a response.

Materials & experimental systems			Methods		
n/a	Involved in the study		n/a	Involved in the study	
☐	☒	Antibodies	☒	☐	ChIP-seq
☐	☒	Eukaryotic cell lines	☐	☒	Flow cytometry
☒	☐	Palaeontology and archaeology	☒	☐	MRI-based neuroimaging
☐	☒	Animals and other organisms			
☒	☐	Human research participants			
☒	☐	Clinical data			
☒	☐	Dual use research of concern			

Antibodies

Antibodies used	Goat anti-CD206 (Santa Cruz; Cat. no. sc-34577, clone: C-20, dilution 1:100)<br>Rabbit anti-CD68 (Santa Cruz, Cat no. sc-9139, clone: H255, dilution 1:100)<br>Rabbit anti-B7-2/CD86 (Novus biologicals, Cat no. NB110-55488, clone: EP1158Y, dilution 1:50)<br>Goat anti-Ym1 (R & D systems, Cat no. BAF2446, dilution 1:50)<br>Rabbit anti-CD36 (Abcam, Cat no. ab133625, clone:EPR6573, dilution 1:50)<br>Rat anti-MHC-II (Santa cruz, Cat no. sc-59318, clone: ER-TR3, dilution 1:100)<br>Donkey Anti-Goat IgG Alexa Fluor® 488 (Life technologies Cat. no. A-11055, dilution 1:100)<br>Donkey anti-Rabbit IgG Alexa Fluor® 488 (Life technologies Cat no. A-21206, dilution 1:100)
Validation	All antibodies have been widely used by the research community and validated by the supplier. A full list with all references to catalog numbers is provided in the suppl. information. Some references with DOI number showing validation of the antibodies or the supplier's webpage are the given below.<br>Goat anti-CD206 (Santa Cruz; Cat. no. sc-34577, clone: C-20): DOI: 10.1007/s11357-020-00299-6<br>Rabbit anti-CD68 (Santa Cruz, Cat no. sc-9139, clone: H255): DOI: 10.1016/j.celrep.2021.109955<br>Rabbit anti-B7-2/CD86 (Novus biologicals, Cat no. NB110-55488, clone: EP1158Y): DOI: 10.3390/ijms222413559<br>Goat anti-Ym1 (R & D systems, Cat no. BAF2446): DOI: 10.1371/journal.ppat.1007423<br>Rabbit anti-CD36 (Abcam, Cat no. ab133625, clone:EPR6573): DOI: 10.1038/s41398-020-01130-8<br>Rat anti-MHC-II (Santa cruz, Cat no. sc-59318, clone: ER-TR3): DOI: 10.1016/j.canlet.2022.215698<br>Donkey Anti-Goat IgG (Life technologies Cat. no. A-11055): https://www.thermofisher.com/antibody/product/Donkey-anti-Goat-IgG-H-L-Cross-Adsorbed-Secondary-Antibody-Polyclonal/A-11055<br>Donkey anti-Rabbit IgG (Life technologies Cat no. A-21206): https://www.thermofisher.com/antibody/product/Donkey-anti-Rabbit-IgG-H-L-Highly-Cross-Adsorbed-Secondary-Antibody-Polyclonal/A-21206

Eukaryotic cell lines

Policy information about cell lines

Cell line source(s)	Mouse macrophages RAW 264.7 and monocytic human THP-1 monocytes - American Type Culture Collection (Rockville, MD)<br>4T1-Luc mouse triple negative breast cancer cells were kindly provided by Dr. O van Tellingen (Netherlands Cancer Institute, Amsterdam, the Netherlands) who received it from Dr. Fred Miller at Karmanos Cancer Institute (Michigan), who originally developed these cells (Miller et al, Invasion Metastasis. 1983, 3 (1): 22-31).<br>CT26 tumor cells were provided by Dr. Hawinkels, Leiden University Medical Centre, The Netherlands, originally obtained from ATCC.
Authentication	We relied on the supplier for the authentication, though we examined the biomarkers using PCR as reported in the manuscript. No cell authentication was used.
Mycoplasma contamination	All cell lines were tested negative for mycoplasma.
Commonly misidentified lines<br>(See ICLAC register)	No commonly misidentified lines were used.

Animals and other organisms

Policy information about studies involving animals; ARRIVE guidelines recommended for reporting animal research

Laboratory animals	6 weeks old female BALB/cAnNRj (18-20g) were purchased from Charles River. Animals were housed in individually ventilated cages and fed ad libitum. The housing temperature was controlled at 20-24°C and humidity of 40-70 % with a 12 h light and 12 h dark cycle was maintained.

Wild animals	The study did not involve wild animals.
Field-collected samples	The study did not involve field-collected samples.
Ethics oversight	The experimental protocol was approved by the Central Animal Ethical Committee, The Netherlands (ethical application numbers: 2013.III.03.024, AVD1100020174305). The work protocol for each experiment was approved by the local Animal Ethical Committee (IvD) of the University of Twente, The Netherlands.

Note that full information on the approval of the study protocol must also be provided in the manuscript.

Flow Cytometry

Plots

Confirm that:

☒ The axis labels state the marker and fluorochrome used (e.g. CD4-FITC).

☒ The axis scales are clearly visible. Include numbers along axes only for bottom left plot of group (a 'group' is an analysis of identical markers).

☒ All plots are contour plots with outliers or pseudocolor plots.

☒ A numerical value for number of cells or percentage (with statistics) is provided.

Methodology

Sample preparation	Tumors were isolated and cut into small pieces and placed in digestion buffer (RPMI containing collagenase II and 0.5 mg/ml of DNAase). The tissue was filtered through a 70 um filter to remove undigested tissue and/or matrix proteins. Cells were fixed, washed with PBS and then blocked with 5% BSA for 1 h. Then, cells were stained with different antibodies against cell surface markers, followed by fluorescently labeled secondary antibodies.
Instrument	BD Biosciences Calibur
Software	Collection: BD CellQuest Pro ver. 5.2.1.<br>Data analysis - Flow Jo ver. 7.6 for in vivo data analysis<br>Flowing software ver. 2.5.0 & 2.5.1 for in vitro data analysis.
Cell population abundance	Cells were not sorted from the tissues but stained to detect specific biomarker in co-localization with nanoliposomes.
Gating strategy	Cells were first gated for intact cells using forward scatter (FSC)/ side scatter (SSC). Cells were then analyzed by cell specific gating using primary antibody, followed by fluorescence-labeled secondary antibodies. During gated, the "negative population" (only secondary antibody) were subtracted. The "positive population" was gated based on the staining for a specific antibody in GFP region while nanoliposomes in RFP region.

☒ Tick this box to confirm that a figure exemplifying the gating strategy is provided in the Supplementary Information.
